# Dual Color Sensors for Simultaneous Analysis of Calcium Signal Dynamics in the Nuclear and Cytoplasmic Compartments of Plant Cells

**DOI:** 10.3389/fpls.2018.00245

**Published:** 2018-02-27

**Authors:** Audrey Kelner, Nuno Leitão, Mireille Chabaud, Myriam Charpentier, Fernanda de Carvalho-Niebel

**Affiliations:** ^1^Laboratory of Plant Microbe Interactions, Université de Toulouse, Institut National de la Recherche Agronomique, Centre National de la Recherche Scientifique, Castanet-Tolosan, France; ^2^Department of Cell and Developmental Biology, John Innes Centre, Norwich, United Kingdom

**Keywords:** *Medicago truncatula*, *Arabidopsis thaliana*, root symbiosis, root hairs, root elongation zone, calcium, GECO sensors, biotic and abiotic stimuli

## Abstract

Spatiotemporal changes in cellular calcium (Ca^2+^) concentrations are essential for signal transduction in a wide range of plant cellular processes. In legumes, nuclear and perinuclear-localized Ca^2+^ oscillations have emerged as key signatures preceding downstream symbiotic signaling responses. Förster resonance energy transfer (FRET) yellow-based Ca^2+^ cameleon probes have been successfully exploited to measure the spatiotemporal dynamics of symbiotic Ca^2+^ signaling in legumes. Although providing cellular resolution, these sensors were restricted to measuring Ca^2+^ changes in single subcellular compartments. In this study, we have explored the potential of single fluorescent protein-based Ca^2+^ sensors, the GECOs, for multicolor and simultaneous imaging of the spatiotemporal dynamics of cytoplasmic and nuclear Ca^2+^ signaling in root cells. Single and dual fluorescence nuclear and cytoplasmic-localized GECOs expressed in transgenic *Medicago truncatula* roots and *Arabidopsis thaliana* were used to successfully monitor Ca^2+^ responses to microbial biotic and abiotic elicitors. In *M. truncatula*, we demonstrate that GECOs detect symbiosis-related Ca^2+^ spiking variations with higher sensitivity than the yellow FRET-based sensors previously used. Additionally, in both *M. truncatula* and *A. thaliana*, the dual sensor is now able to resolve in a single root cell the coordinated spatiotemporal dynamics of nuclear and cytoplasmic Ca^2+^ signaling *in vivo*. The GECO-based sensors presented here therefore represent powerful tools to monitor Ca^2+^ signaling dynamics in *vivo* in response to different stimuli in multi-subcellular compartments of plant cells.

## Introduction

Divalent calcium (Ca^2+^) ions are versatile second messengers regulating many biological processes in eukaryotic and prokaryotic cells (Dodd et al., 2010; Domínguez et al., 2015). In plants, Ca^2+^ mediates developmental and physiological responses to various biotic and abiotic environmental cues (McAinsh and Pittman, 2009; Dodd et al., 2010; Kudla et al., 2010; Stael et al., 2012). Stimuli-induced Ca^2+^ releases result in transient or repetitive oscillations, distinct in their frequency, amplitude, duration, and spatial distribution. The sensing and decoding of these so-called Ca^2+^ signatures (McAinsh and Pittman, 2009) by Ca^2+^-binding proteins assure the transduction of the signal to downstream transcriptional and metabolic responses (Galon et al., 2010; Reddy et al., 2011; Whalley and Knight, 2013).

The analysis of Ca^2+^ signatures and their contribution to signaling in different cellular compartments requires tools adapted for monitoring Ca^2+^ dynamics with high spatial and temporal resolution. Bioluminescent aequorin-based probes can successfully quantify variations in Ca^2+^ signaling in plants, but their low light emission limits the spatiotemporal resolution of these responses during imaging (Knight et al., 1991; McCombs and Palmer, 2008). Cellular resolution can be achieved by the use of fluorescent dyes and genetically-encoded Ca^2+^ indicators (GECIs), consisting of fluorescent proteins (FPs) fused to sensor domains. Compared to fluorescent dyes, GECIs have the advantage of being accessible to any cell type and offer the possibility of measuring Ca^2+^ variations in subcellular compartments, by specific targeting of the sensor. The most popular GECI sensors are the ratiometric Förster Resonance Energy Transfer (FRET)-based Yellow Cameleons (YCs) composed of a fluorescent donor-acceptor pair, respectively, CFP and YFP, linked by a calmodulin (CaM) and the CaM-binding peptide of myosin light-chain kinase M13 (Miyawaki et al., 1997; Allen et al., 1999). Upon Ca^2+^ binding to CaM, CaM and M13 interact which triggers protein conformational changes that result in increased FRET between the donor-acceptor pair. More recently, improved FRET-based pairs or hybrid sensors such as BRET (bioluminescent aequorin coupled with a FP) have been engineered and successfully used in plants (Rodriguez-Garcia et al., 2014; Xiong et al., 2014; Bajar et al., 2016).

Targeting FRET-based probes to different subcellular compartments revealed precise Ca^2+^ variations in different organelles, including the nucleus, the endoplasmic reticulum, the chloroplast, peroxisomes, and mitochondria (Iwano et al., 2009; Sieberer et al., 2009; Costa et al., 2010; Krebs et al., 2012; Loro et al., 2012, 2016; Stael et al., 2012; Bonza et al., 2013). The Ca^2+^ dynamics in specific compartments are likely to reflect the differential activation of Ca^2+^ channels and the contribution of diverse intra and extracellular Ca^2+^ stores in the control of specific functions, such as gene transcription (Ranty et al., 2012). A number of stimuli activates Ca^2+^ responses simultaneously in different subcellular compartments (van Der Luit et al., 1999; Wood et al., 2001). As such, in legumes, the perception of diffusible nodulation (Nod) and mycorrhizal (Myc) factors released by nitrogen-fixing bacteria and arbuscular mycorrhiza (AM), fungi respectively, induces calcium oscillations in the nucleus and adjacent cytoplasm (Ehrhardt et al., 1996; Miwa et al., 2006a; Sieberer et al., 2009). Symbiotic calcium spiking was monitored using fluorescent dyes, cytoplasmic FRET-based YC probes (Ehrhardt et al., 1996; Catoira et al., 2000; Wais et al., 2000; Shaw and Long, 2003; Miwa et al., 2006a,b; Kosuta et al., 2008) or nuclear-tagged YFP-FRET probes (Sieberer et al., 2009, 2012; Chabaud et al., 2011; Genre et al., 2013; Sun et al., 2015). Although monitored independently, cytoplasmic and nuclear Ca^2+^ responses were hypothesized to be synchronized based on their relative time period. Nod factors (NF), consisting of lipo-chitooligosaccharides, and AM fungi chitin oligomers (COs), both induce Ca^2+^ spiking, essential for the transduction of downstream symbiotic signaling in the nucleus (Zipfel and Oldroyd, 2017). More recently, major components involved in the generation of Ca^2+^ spiking were identified in legumes. In the model legume *Medicago truncatula*, the potassium channel DMI1 (Ané et al., 2004), the Ca^2+^-ATPase MCA8 (Capoen et al., 2011), and CNGC15 Ca^2+^ channels (Charpentier et al., 2016), are required for the generation of symbiotic Ca^2+^ oscillations (Granqvist et al., 2012; Charpentier and Oldroyd, 2013; Charpentier et al., 2016). Interestingly, they all locate to both the outer nuclear membrane (ONM) and the inner nuclear membrane (INM) (Capoen et al., 2011; Charpentier et al., 2016). This observation raises the issue of the direction of the observed Ca^2+^ release. Ca^2+^ could be simultaneously released across the INM and the ONM or, sequentially, through the INM or the ONM to the nucleoplasm and cytoplasm, respectively. Thus, simultaneous investigation of nuclear and cytoplasmic Ca^2+^ dynamics is required to gain information on the origin of the specific symbiotic factor-induced nuclear Ca^2+^ signals, and clarify the spatial activation of the ion channels.

Recent studies in animal cells revealed that highly sensitive Ca^2+^ GECI sensors can discriminate stimulus-dependent directional propagation of Ca^2+^ signals (Nakao et al., 2015). These improved GECIs, called GECOs (for GECIs for optical imaging) are circularly permuted single fluorescent intensiometric Ca^2+^-sensing proteins with a CaM and a M13 peptide domains. Upon Ca^2+^ binding, changes in protein conformation result in a visible increase in its fluorescence intensity (Zhao et al., 2011). GECOs have to date only been targeted to the cytoplasm to study Ca^2+^ signaling in plant cells (Ngo et al., 2014; Keinath et al., 2015; Wang et al., 2015; Tunc-Ozdemir and Jones, 2017; Waadt et al., 2017). However, the available palette of GECO fluorescent protein colors now opens the possibility of concomitantly imaging Ca^2+^-signaling in different compartments of plant cells (Zhao et al., 2011). Moreover, improved GECO versions were generated through directed evolution and screened for higher intensity signal change upon Ca^2+^ binding, but not yet used in plants (Zhao et al., 2011; Wu et al., 2013).

In this study, we generated dual Ca^2+^ sensors that simultaneously monitor the Ca^2+^ dynamics in the nucleus and cytoplasm, by targeting highly sensitive GECO Ca^2+^ sensors to these subcellular compartments. We demonstrated that the new dual GECO sensors are powerful tools to concomitantly monitor *in vivo* Ca^2+^ dynamics in adjacent nuclear and cytoplasmic compartments of both *M. truncatula* and Arabidopsis roots in response to both biotic and abiotic signals. We unraveled that the symbiotic Nod factor-induced nuclear calcium oscillations preferentially start in the nucleus. Additionally the dual GECO sensor revealed that biotic and abiotic stimuli trigger distinct cytoplasmic and nuclear Ca^2+^ dynamics in *A. thaliana* root cells.

## Results

### The highly sensitive GECO sensor enables real-time visualization of nuclear Ca^2+^ spiking in *M. truncatula*

In order to evaluate the performance of GECO as a Ca^2+^ sensor for the symbiotic factor-induced nuclear Ca^2+^ oscillations in *M. truncatula*, we designed a compatible binary vector to constitutively express the red fluorescence R-GECO1 (Zhao et al., 2011) in the nucleus of plant cells (Figure S1). This construct, referred to as NR-GECO1, comprises a viral SV40 nuclear localization signal (NLS) fused to the R-GECO1 and driven by the double *CAMV 35S* promoter (*2xp35S*) (Figure S1), and was used together with the previously characterized yellow fluorescence cameleon sensors NUP-YC2.1 (Sieberer et al., 2009) and NLS-YC3.6 (Krebs et al., 2012) to generate *M. truncatula* composite plants via *Agrobacterium rhizogenes*-mediated transformation. Confocal laser scanning microscopy (CLSM) revealed that both the NR-GECO1 and the two cameleon sensors were targeted to the nuclear compartment of *M. truncatula* root cells (Figure S2). NR-GECO1 fluorescence was undetectable in the cytoplasm, and appeared homogeneously distributed within the nucleus (Figure S2A).

**Figure 1:**
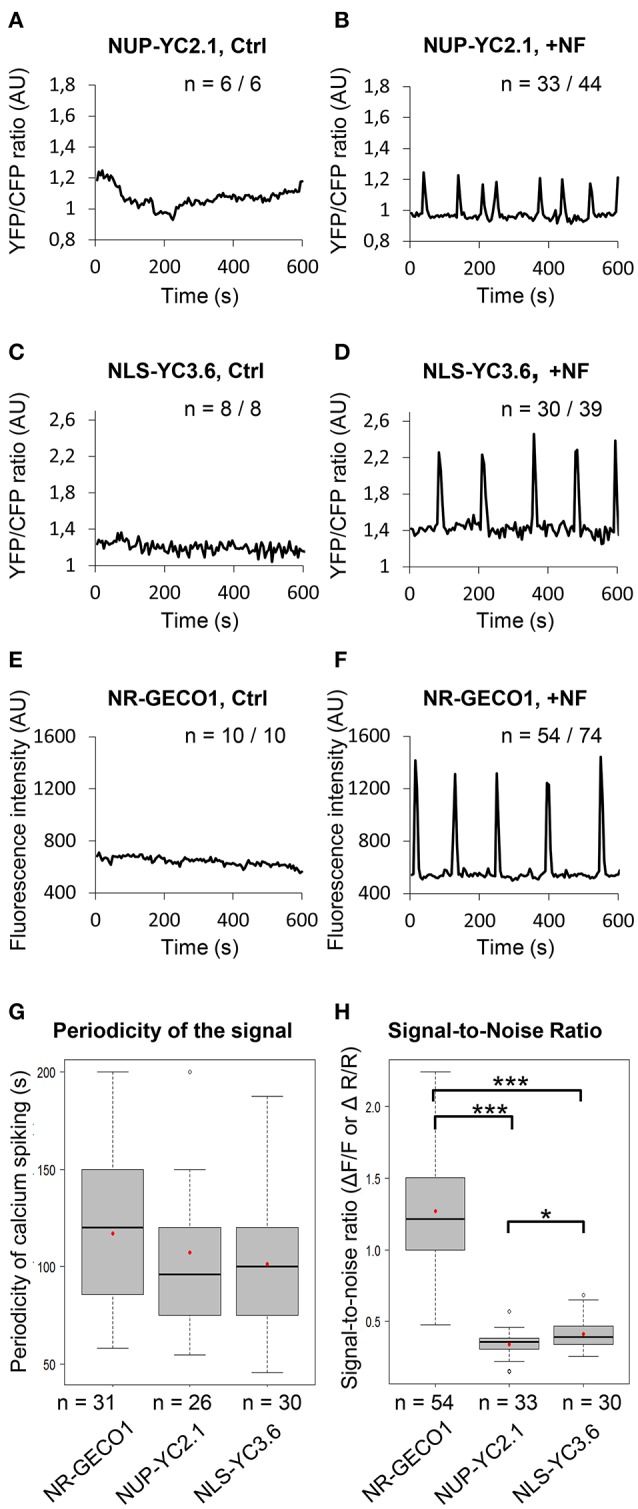
NR-GECO1 displays higher Ca^2+^ signal-to-noise ratio than previously used cameleon sensors. **(A–F)** Representative profiles obtained with the FRET probes NUP-YC2.1 **(A,B)**, NLS-YC3.6 **(C,D)**, and the single NR-GECO1 sensor **(E,F)** before (Ctrl) and after Nod Factor (+NF) addition. *n* represents the number of individual root hair cells analyzed from 2 to 3 independent experiments (AU, Arbitrary Units). Note that the y-axis scales are different for each sensor. **(G,H)** Calcium spiking periodicity **(G)** and signal-to-noise ratio **(H)** was calculated for each individual sensor. *in vivo* Signal-to-noise ratio (SNR) in **(H)** represents signal amplitude changes during calcium spiking and was calculated using fluorescence intensity values (ΔF/F) for NR-GECO1 and YFP/CFP ratio (ΔR/R) values for the cameleon sensors. Box plots represent first and third quartile (horizontal box sides), minimum and maximum (outside whiskers), median (central lines), and mean (solid red circle) values. A one-way ANOVA followed by a Tukey honest significant (HSD) test of the values after a Box-Cox transformation (λ = 0.0606) did not reveal statistical differences between the three groups (*p* > 0.05) in **(G)**. A Kruskal-Wallis test revealed statistical difference between the groups in **(H)** (^*^p < 0.05 and ^***^p < 0.001).

Symbiosis-related nuclear Ca^2+^ responses were assessed in the nucleus of growing root hair cells using CLSM (Figure S3). The basal fluorescence signal intensity was recorded for 5–10 min for each sensor before treatment of roots with purified *Sinorhizobium meliloti* NFs (10^−9^ M) (Figures 1A,C,E). Following NF application, sustained Ca^2+^ spikes were observed within the nucleus of cells transformed with the three sensors (Figures 1B,D,F). They all generated comparable patterns of NF-induced Ca^2+^ spiking, with spike periodicity of about 100–120 s (Figure 1G).

To evaluate whether NR-GECO1 was suitable for detecting other Ca^2+^ responses in *M. truncatula*, we challenged *M. truncatula* composite plants and root organ cultures (ROCs) with tetra-chitooligosaccharides (CO4) previously shown to elicit nuclear Ca^2+^ oscillations (Genre et al., 2013; Sun et al., 2015). Similarly to NFs, NR-GECO1 allowed the detection of CO4-induced nuclear Ca^2+^ spiking in both composite plants and ROCs (Figure S4). As previously observed with a cameleon probe (Genre et al., 2013), the NF receptor *nfp* mutant no longer exhibited NF-elicited Ca^2+^ spiking but still displayed CO4-induced Ca^2+^ spiking responses (Figures S4E–G). Taken together, NR-GECO1 can efficiently measure symbiosis-related nuclear Ca^2+^ spiking responses in *M. truncatula* roots.

To investigate sensor performance, we compared the maximum signal change to the basal fluorescence signal (before the spike), by calculating the signal-to-noise ratio (SNR) values for each individual probe. The fractional fluorescence changes (ΔF/F) for NR-GECO1 and the fractional ratio changes (ΔR/R) for the cameleon sensors were measured as described (Figure S3B and Material and Methods), from a high number of individual root hairs. FRET-based probes exhibited rather similar dynamic changes, although NLS-YC3.6 appeared to be slightly superior to NUP-YC2.1 with mean SNR values of 0.41 and 0.34, respectively (Figure 1H). However, there was a striking difference in the dynamic responses observed between the nuclear NR-GECO1 and the cameleon sensors with a mean SNR value of 1.27 (Figure 1H). Thus, NR-GECO1 allows monitoring of NF-induced nuclear Ca^2+^ spiking with a greater sensitivity than the cameleon sensors. Our results confirm the previously described higher sensitivity of the cytoplasmic R-GECO1 in comparison to the YC3.6, in response to the ATP-induced Ca^2+^ signals in *A. thaliana* roots (Keinath et al., 2015). Additionally, using CLSM, NR-GECO1 allowed real time visualization of the Ca^2+^ changes in contrast to the cameleon probe for which the YFP/CFP fluorescent ratio have to be calculated from a pre-selected region of interest (ROI) (Figure S5 and Movies S1, S2). Thus, in comparison to cameleon sensors, NR-GECO1 has a higher dynamic range, which greatly facilitates the rapid monitoring of cell-autonomous dynamics of Ca^2+^ spiking in various *M. truncatula* root hair and non-hair cells.

### A dual GECO sensor for simultaneously monitoring Ca^2+^ signals in both nuclear and cytoplasmic compartments of *M. truncatula* root cells

**Figure 2:**
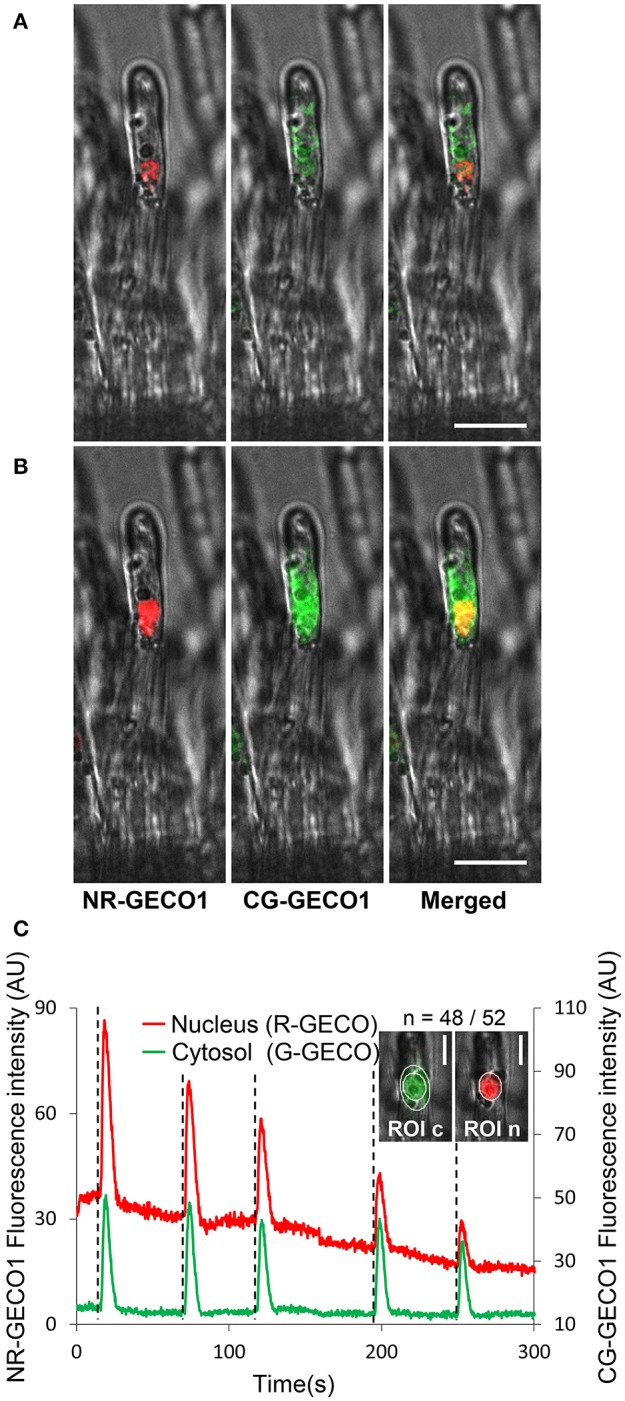
A dual GECO Ca^2+^ sensor targeted to both nuclear and cytoplasmic subcellular compartments reveals coordinated oscillatory Ca^2+^ responses. **(A,B)** Subcellular localization of the dual NRCG-GECO1 sensor in *M. truncatula* root hair cells. Confocal fluorescent images of NRCG-GECO1 sensor before **(A)** and during a spike **(B)** are represented as separated red, green, and merged images with respective bright fields. **(C)** Relative fluorescence intensity traces of NR-GECO1 (red) and CG-GECO1 (green) at 0.25 s intervals from ROIs selected around the nuclear (ROI n) and peri-nuclear cytoplasmic (ROI c) regions of a *M. truncatula* root hair treated with a 10^−9^ M Nod Factor (+NF) solution. Dotted lines mark the initiation of a spike, *n* corresponds to the number of root hairs responding positively/total number of analyzed root hairs. Data were collected from two biological experiments. Scale bars represent 20 μm **(A,B)** and 10 μm **(C)**.

Taking advantage of the high sensitivity of GECOs, we generated a dual fluorescence color sensor for imaging Ca^2+^ signal dynamics simultaneously in the cytoplasm and the nuclear compartments of the same root hair cell. We therefore combined the nuclear-tagged red GECO1 and the cytoplasmic green GECO1 in a single construct (Figure S1). In this dual sensor, both the nuclear red and cytoplasmic green GECOs (NRCG-GECO1) are driven by *2xp35S* promoters. CLSM imaging revealed the dual labeling of nuclear and cytoplasmic compartments of *M. truncatula* epidermal cells (Figure 2A). In a non-spiking state (Figure 2A), the red and green fluorescence proteins are found, respectively, in the nuclear and cytoplasmic regions demonstrating the correct targeting of the GECO to the expected subcellular compartments. However, during a Ca^2+^ spike a merged orange-to-yellow fluorescence signal is observed in the nuclear region, due to the acquisition of increased nuclear R-GECO1 and perinuclear green G-GECO1 fluorescence signals (Figure 2B).

Imaging conditions were set up to avoid crossover between fluorescence emissions of the two probes. The excitation of G-GECO1 and R-GECO1 is achieved using an argon 488 nm laser and a 561 nm diode, respectively, and emission is detected in the respective fluorescence window ranges of 500–550 and 580–643 nm (Zhao et al., 2011). By using the optimum excitation parameters for G-GECO1 (488 nm excitation, Figure S6A), the green cytoplasmic fluorescence was clearly observed in the expected 500–550 nm emission window in NF-treated *M. truncatula* root hairs. However, a slight bleed-through of the G-GECO1 was observed in the R-GECO1 emission window of 580–643 nm protein. To avoid this, we therefore reduced the fluorescence window emission range for R-GECO1 from 580–643 to 600–643 nm. Importantly, no bleed-through of the R-GECO1 was observed in the G-GECO1 500–550 nm emission window. Thus, the dual NRCG-GECO1 sensor expressed in transformed roots of *M. truncatula* composite plants was able to monitor concomitant nuclear and cytoplasmic Ca^2+^ spiking in an individual NF-treated root hair (Figure 2C). Similar coordinated Ca^2+^ responses in the two subcellular compartments were monitored in *M. truncatula* transgenic roots treated with CO4 (Figure S7A). To ensure the specificity of these responses, we used the dual NRCG-GECO1 sensor in the *dmi1* and *dmi3* mutants (Catoira et al., 2000; Arrighi et al., 2006). As expected, coordinated NF-elicited Ca^2+^ spiking was only observed in the *dmi3* mutant (Figure S7). Taken together, the dual NRCG-GECO1 Ca^2+^ sensor targets GECO fluorescent proteins to two subcellular compartments and is suitable for co-imaging of specific Ca^2+^ responses in real time in two distinct subcellular compartments without fluorescence bleed-through.

### Ca^2+^ spiking preferentially initiates in the nucleus of medicago root hairs responding to symbiotic nod factors

**Figure 3:**
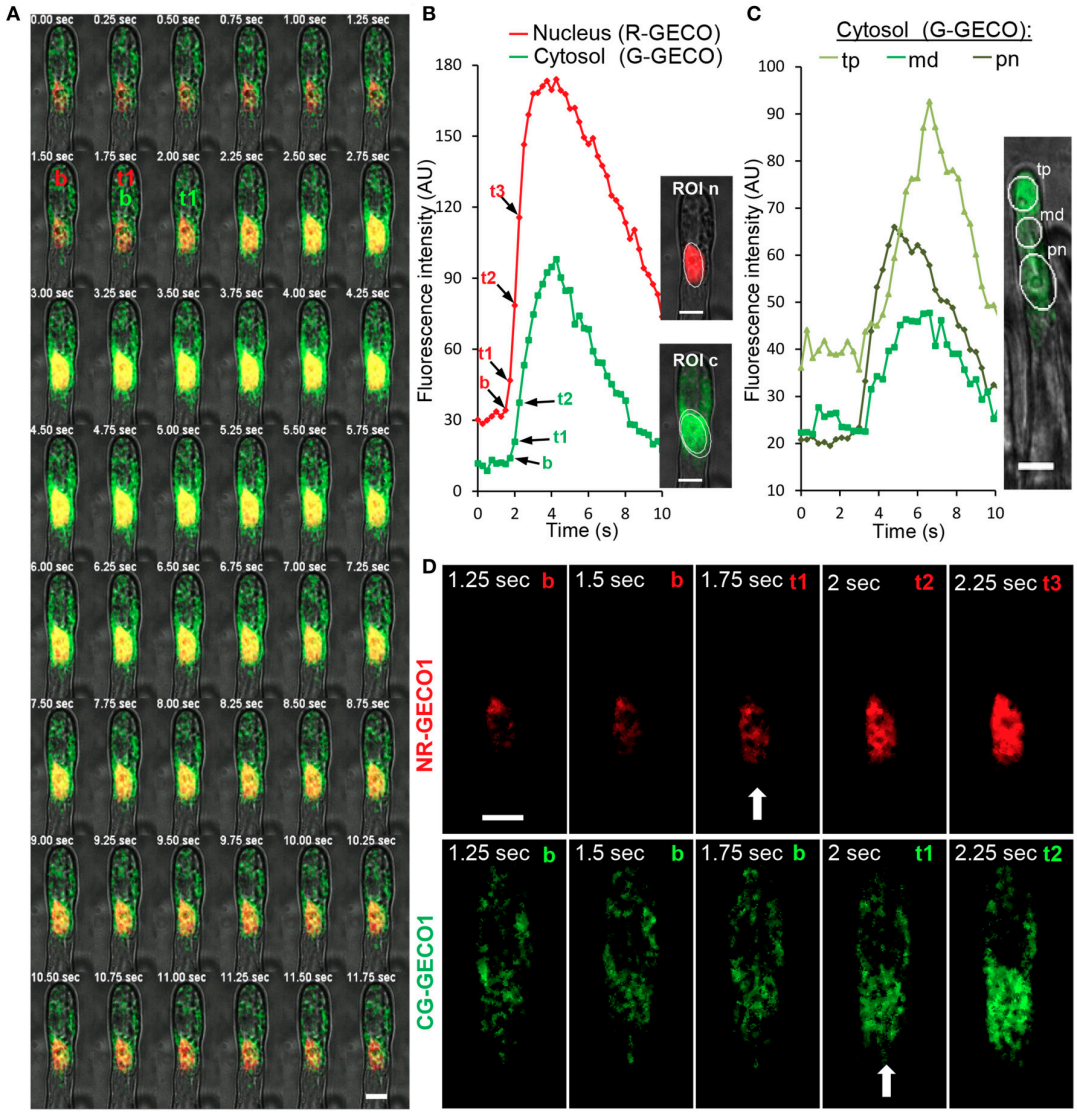
Spatiotemporal dynamics of Ca^2+^ spiking in nuclear and cytoplasmic compartments of a *M. truncatula* root hair responding to NFs. **(A)** A sequential frame-by-frame co-imaging (0.25 s intervals) of nuclear-targeted NR-GECO1 and cytosolic CG-GECO1 revealed coordinated responses during a single Ca^2+^ spike. **(B)** Relative fluorescence intensity traces of NR-GECO1 (red) and CG-GECO1 (green) from ROIs selected around the nuclear (ROI n) and perinuclear cytoplasmic (ROI c) regions. Images on the right represent the maximum projection of respective fluorescence images merged with bright field. **(C)** Relative fluorescence intensity (0.3 s intervals) of CG-GECO1 in selected cytoplasmic regions of interest in the perinuclear (pn), middle (md), or root hair tip (tp) regions during a NF-triggered Ca^2+^ spike. **(D)** Close view of selected fluorescence frames shown in **(A)**. Time points representing baseline fluorescence [b in **(A,B,D)**], the start of the spike [t1 and arrow **(A,B,D)**], and subsequent spike rise time points [t2 and t3 in **(A,B,D)**] are indicated for NR-GECO1 and CG-GECO1. Scale bars represent 10 μm.

**Figure 4:**
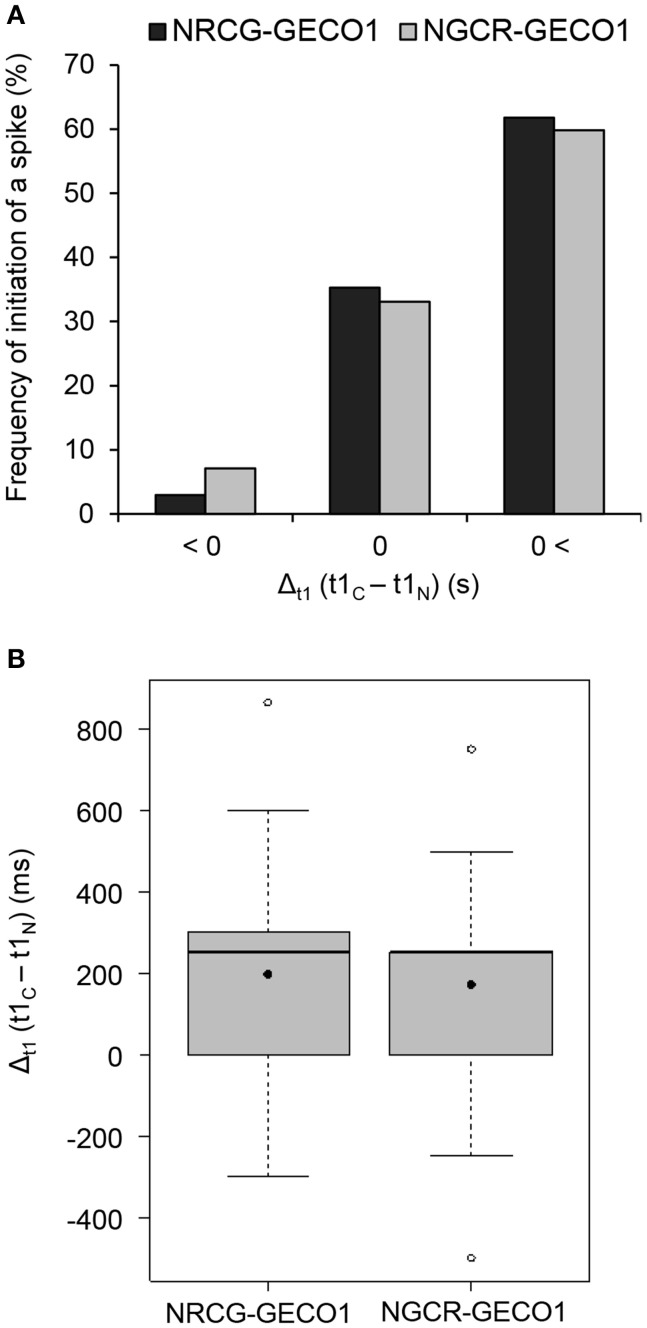
Monitoring NF-elicited Ca^2+^ signaling with dual GECO sensors reveals a preferential Ca^2+^ spike start in the nuclear compartment. Time values corresponding to the start of individual spikes (t1, illustrated in Figure 3B) were collected for each sensor and used to calculate the relative delay in the start of a spike. The time interval (Δ_t1_ in s), between peak start measured by sensors, was calculated by subtracting nuclear t1 from cytoplasmic t1-values (t1_C_-t1_N_). A Δ_t1_ value below 0 indicates a cytoplasmic-to-nucleus spike start (C → N), a Δ_t1_-value close to 0 indicates coordinated cytoplasmic and nuclear spike start (C = N), and a positive Δ_t1_-value indicates a nucleus-to-cytoplasmic spike start (C←N). These analyses were done with *n* = 68 (NRCG-GECO1) and *n* = 127 (NGCR-GECO1) individual spikes from 26 or 24 individual nuclei, respectively. **(A)** Frequency of the individual spikes showing C → N, C = N, and C←N patterns in the dual NRCG-GECO1 (dark gray) and NGCR-GECO1 (light gray) sensors. No significant differences were found between the sensors (*p* > 0.05, chi-square test of goodness-of-fit). Frequency distribution within the different categories (C → N, C = N, and C←N), for each dual sensor, is significantly different and not due to a random distribution (1/3 of observations in each category, *p* < 0.001, chi-square test of goodness-of-fit). **(B)** Time interval between spike start (Δ_t1_ in ms) measured for each dual sensor. Average/median values for NRCG-GECO1 and NGCR-GECO1 of 197/250 and 174/250 ms, respectively, are not significantly different. Box plots represent first and third quartile (horizontal box sides), minimum and maximum (outside whiskers), median (central lines), and mean (solid black circle).

The dual sensor made it possible to simultaneously visualize highly coordinated Ca^2+^ signals in both nuclear and cytoplasmic compartments and therefore resolve the initiation dynamics of the symbiotic factor-induced nuclear Ca^2+^ spiking. To increase temporal resolution, we reduced the image acquisition intervals to below 0.5 s. A qualitative frame-by-frame analysis illustrated the rise in both red and green fluorescence of respective GECOs during a single spike (Figure 3A). The selection of regions of interest (ROI) in the nucleoplasm and in different cytoplasmic-associated areas allowed us to precisely follow the progressive rise in fluorescence intensity of the sensors over time (Figures 3B,C). In both compartments, the start of a Ca^2+^ spike was recognized by a steep increase in fluorescence intensity to baseline fluorescence levels (Figures 3B–D). These analyzes revealed a strong Ca^2+^ rise in the nucleus and in the nuclear periphery, followed by sequential signal propagation toward the root hair tip (Figures 3B,C). To further evaluate the dynamics of Ca^2+^ spiking initiation in the perinuclear region, we simultaneously recorded fluorescence data from nuclear NR-GECO1 and cytoplasmic CG-GECO1 from delimited ROI zones (Figure 3B) of individual NF-treated root hairs. The precise time for peak initiation (t1) for both GECO probes was compared by calculating the Δ_t1_ value (CG-GECO1_t1_-NR-GECO1_t1_) for a large number of individual root hairs (Figure 4A). These analyses revealed that the majority of Ca^2+^ spiking (65%) initiated primarily and significantly in the nuclear compartment, while only 2% initiated first in the cytosol. In 30% of the cases, it was not possible to clearly distinguish differences between the Ca^2+^ spiking initiation sites.

To exclude intrinsic differences between the sensors in the targeted compartments we generated a reverse dual sensor expressing R-GECO1 in the cytoplasm and the G-GECO1 in the nucleus (Figures S1, S8). Using the dual NGCR-GECO1 sensor we confirmed the preferential initiation of Ca^2+^ spiking in the nuclear compartment (Figure 4A). The time interval (Δ_t1_) for the initiation of a spike between the nuclear and cytoplasmic compartments was similar for both dual sensors (Figure 4B), with median values of 250 ms. Taken together, the dual GECO1 Ca^2+^ sensors generated here allowed us to measure coordinated Ca^2+^ responses in different subcellular compartments of individual cells (Movies S3, S4) and to demonstrate that Ca^2+^ spiking primarily initiates in the nuclear compartment of root hair cells responding to rhizobial Nod factors.

### Ca^2+^ signal dynamics in both nuclear and cytoplasmic compartments in *A. thaliana* root elongation zone cells

To investigate the use of the dual sensor in plant species other than *M. truncatula*, we stably transformed *A. thaliana* with the NRCG-GECO1.2 sensor (Figure S1) comprising improved versions of R-GECO1 and G-GECO1 (Zhao et al., 2011; Wu et al., 2013). Stable transgenic lines were propagated until the T3 generation under hygromycin selection and selected for expression of the dual sensor. CLSM imaging of *A. thaliana* roots demonstrated that CG-GECO1.2 and NR-GECO1.2 localized to the cytoplasmic and nucleoplasmic compartments, respectively (Figure S9). The stable transgenic line is phenotypically like wild type plants, indicating that the constitutive expression of the sensor does not interfere with cellular Ca^2+^ signaling and homeostasis (Figure S9). In *A. thaliana* roots transformed with aequorin or cameleon YC3.6, several studies have reported that biotic and abiotic stimuli induce Ca^2+^ release. As such, chitin, NaCl, ATP, and cold stress have been shown to induce Ca^2+^ release in the root elongation zone (Kiegle et al., 2000; Tanaka et al., 2010; Keinath et al., 2015). However, the subcellular dynamics of these stimuli-induced Ca^2+^ releases is lacking.

**Figure 5:**
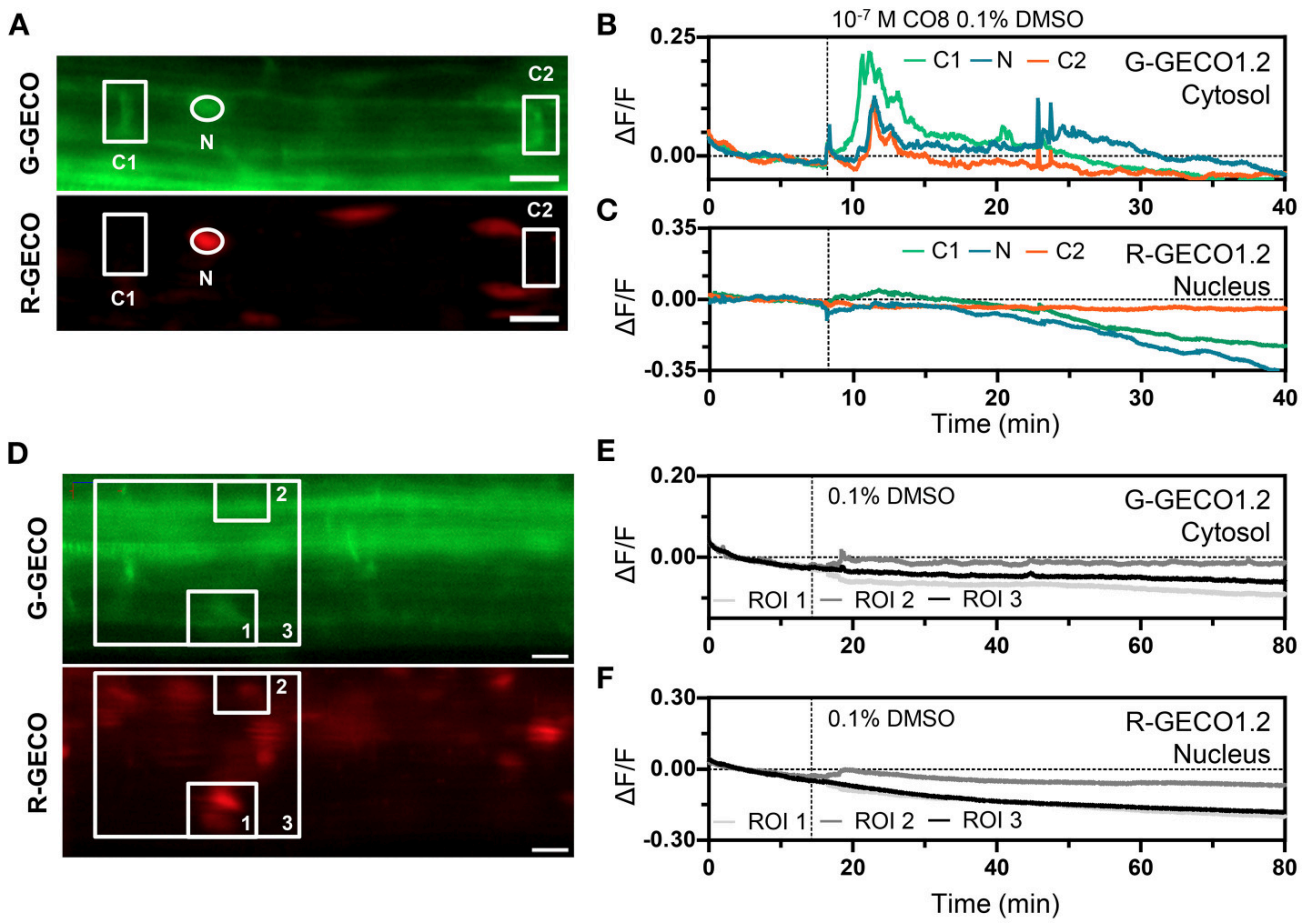
The dual Ca^2+^ sensor reports Ca^2+^ dynamics to CO8. **(A–C)** Representative Ca^2+^ signals induced by 10^−7^ M CO8 in a 5-day-old Arabidopsis root (elongation zone) expressing the dual Ca^2+^ reporter NRCG-GECO1.2 [**(A)**, G-GECO1.2 channel (top); R-GECO1.2 channel (bottom)]. **(B,C)** Normalized fluorescence intensities in the G-GECO1.2 channel **(B)** and the R-GECO1.2 channel **(C)** of the ROIs marked in **(A)** (*n* = 8/11 plants). **(D,F)** 0.1% DMSO did not induce Ca^2+^ signals in a 5-day-old root (elongation zone) expressing the dual Ca^2+^ reporter NRCG-GECO1.2 [**(D)**, G-GECO1.2 channel (top); R-GECO1.2 channel (bottom)]. **(E,F)** Normalized fluorescence intensities in the G-GECO1.2 channel **(E)** and the R-GECO1.2 channel **(F)** of the ROIs marked in **(D)** (*n* = 4 plants). Dashed vertical lines mark the moment of CO8 and DMSO application. Scale bars represent 20 μm.

To monitor the dynamics of cytoplasmic vs. nucleoplasmic Ca^2+^ release in response to chitooctaose (CO8), NaCl, ATP and cold stress, we focused our study on the root epidermal cells of the elongation zone. Treating 5 day-old roots with CO8 to a final concentration of 10^−7^ M elicited an increase in cytoplasmic Ca^2+^ within 2 min of application in 73% of the plants tested (8/11 plants) (Figures 5A–C). Ca^2+^ release was detected only in the cytoplasmic region without any propagation to the nucleoplasm. The Ca^2+^ signature was characterized by a sharp increase followed by progressive smaller oscillations returning to baseline levels over a period of 15 min (Figure 5B). The Ca^2+^ signal was not detected after treatment with the buffer control (Figures 5D–F). Similarly, ATP to a final concentration of 100 μM induced a specific cytoplasmic Ca^2+^ release 7 min after application (Figure S10).

**Figure 6:**
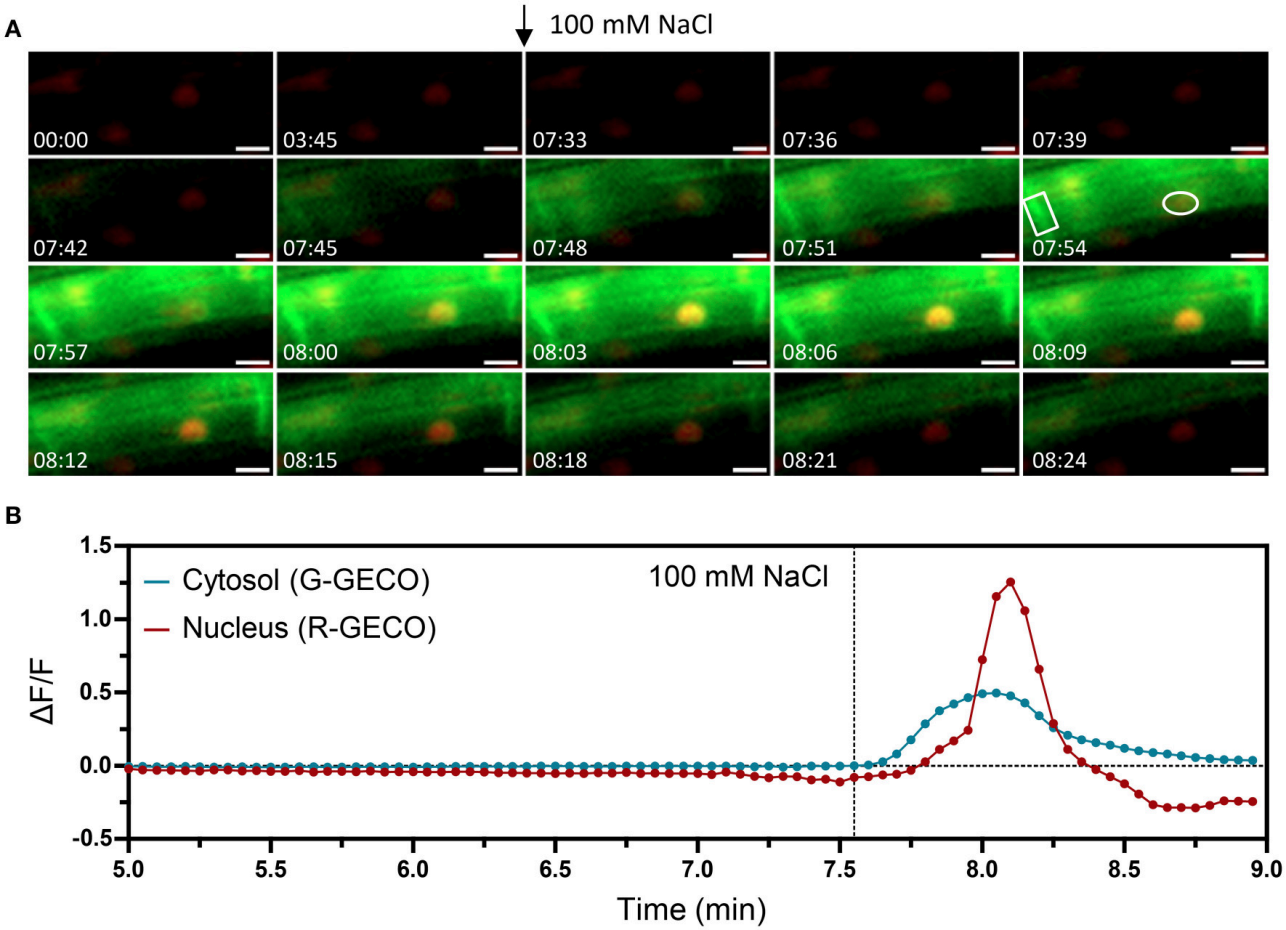
Nuclear and cytosolic Ca^2+^ signals in response to 100 mM NaCl. Ca^2+^ signals induced by salt stress in a 6-day-old Arabidopsis root (elongation zone) expressing the dual Ca^2+^ reporter NRCG-GECO1.2. **(A)** Time-lapse images overlaying the G-GECO1.2 channel (green) and the R-GECO1.2 (red) showing the progression of the salt-induced signal through a cell (timestamps are in minutes and seconds and treatment application is marked with an arrow). **(B)** Normalized fluorescence intensities in the cytosol quantified in the G-GECO1.2 channel (blue) and in the nucleus quantified in the R-GECO1.2 channel (red). The areas quantified are marked in **(A)**. Dashed vertical line marks the moment of treatment with NaCl to a final concentration of 100 mM (*n* = 7/8 plants). Scale bars represent 10 μm.

**Figure 7:**
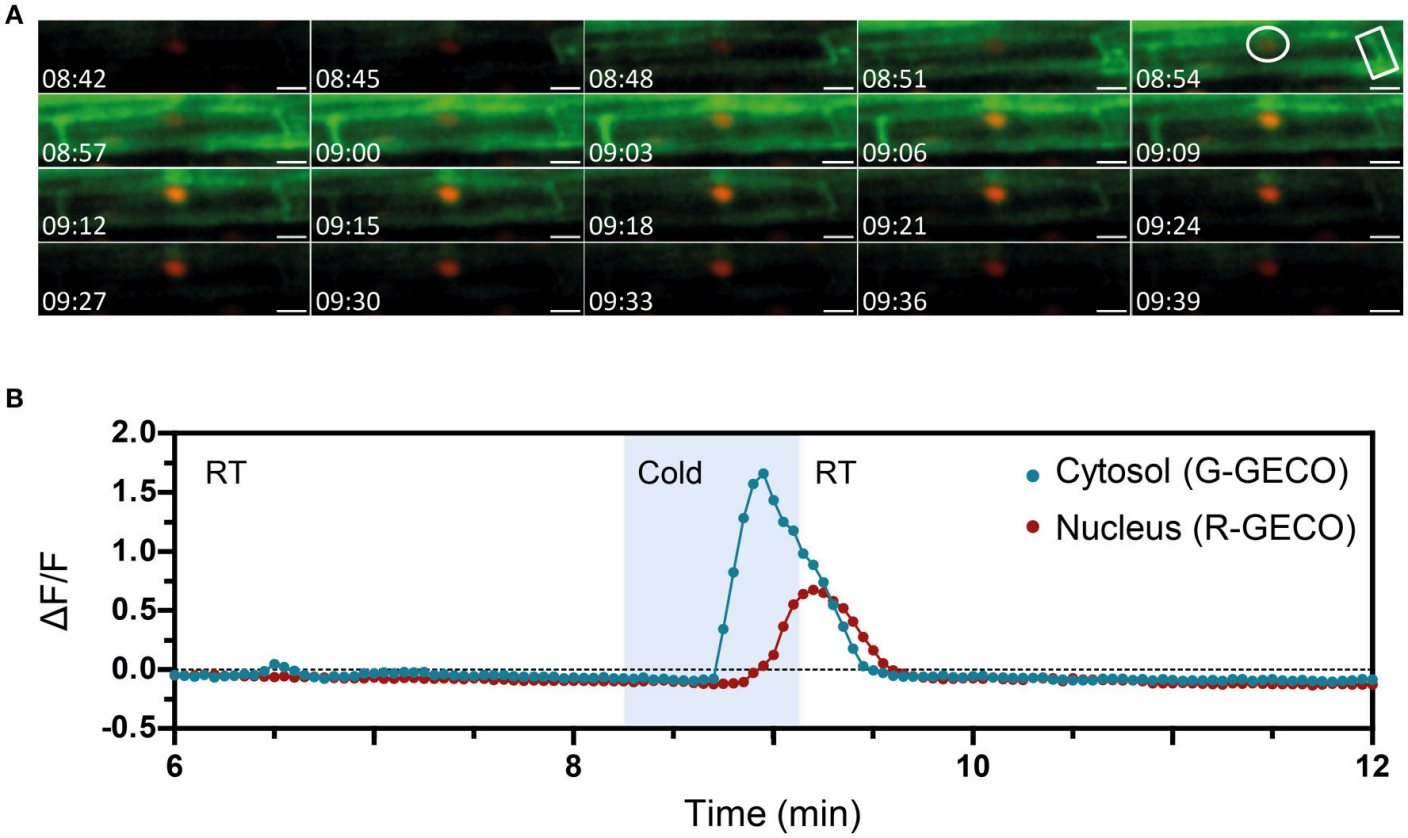
The nuclear and cytosolic Ca^2+^ signals in response to cold are distinct. Ca^2+^ signals induced by cold treatment in a 6-day-old Arabidopsis root (elongation zone) expressing the dual Ca^2+^ reporter NRCG-GECO1.2. **(A)** Time-lapse images overlaying the G-GECO1.2 channel (green) and the R-GECO1.2 (red) showing the progression of the cold-induced signal through a cell (timestamps are in minutes and seconds). Cold was perfused between 08:15 and 09:09. **(B)** Normalized fluorescence intensities in the cytosol quantified in the G-GECO1.2 channel (blue) and in the nucleus quantified in the R-GECO1.2 channel (red). The areas quantified are marked in **(A)**. Area shaded in blue corresponds to the period of cold perfusion (*n* = 4 plants). Scale bars represent 50 μm.

The ATP-induced Ca^2+^ signature was similar to the CO8-induced Ca^2+^ release with a sharp peak followed by smaller oscillations, although their frequencies differed (0.6 min^−1^ for CO8 and 1.94 min^−1^ in the case of ATP) (Figure S10). In contrast to CO8 and ATP, which induced a specific cytoplasmic Ca^2+^ response with an oscillatory pattern, NaCl and cold induced Ca^2+^ release in both the cytoplasmic and nucleoplasmic compartments (Figures 6, 7, Movies S5, S6). Each of these stimuli induced a transient Ca^2+^ release first in the cytoplasm followed by a transient Ca^2+^ increase in the nucleoplasm (Figures 6, 7). However, the dynamics of each nucleoplasmic Ca^2+^ increase differ in their amplitudes and timing of activation. Cold shock induced a transient nucleoplasmic Ca^2+^ release 20 to 30 s after the cytoplasmic Ca^2+^ response (Figure 7). In contrast, NaCl treatment induced a Ca^2+^ response in the nucleus 2–3 s after cytoplasmic Ca^2+^ release (Figure 6). Additionally, the amplitude of NaCl-induced nucleoplasmic Ca^2+^ release is higher than the cytoplasmic Ca^2+^ release, which contrasts with the cold-induced nucleoplasmic Ca^2+^ signal, which is lower than the cytoplasmic Ca^2+^ signal. The differences in timing of activation and amplitude of the Ca^2+^ signals between both compartments strongly suggest that cold and NaCl induce distinct cytoplasmic and nucleoplasmic Ca^2+^ signals. The nucleoplasmic response might be a consequence of the cytoplasmic Ca^2+^ release, or induced independently. All together our results demonstrate that the dual sensor can effectively monitor Ca^2+^ dynamics simultaneously in the cytoplasm and nuclear regions in *A. thaliana* roots cells, and identify various Ca^2+^ signatures generated in response to biotic and abiotic stimuli.

## Discussion

Recent years have witnessed powerful advances in genetically-encoded fluorescent Ca^2+^ sensors, notably with the development of single fluorescent protein GECIs, the GECOs (Zhao et al., 2011). These sensors were engineered to emit fluorescence at different wavelengths, thus enabling multicolor and multiparameter imaging in eukaryotic cells (Zhao et al., 2011; Ngo et al., 2014; Odaka et al., 2014; Waadt et al., 2017). Moreover, the GECOs have a higher sensitivity compared to the FRET-based cameleon YC3.6 (Keinath et al., 2015), which we confirmed in response to symbiotic factors in *M. truncatula* roots. The availability of multicolor Ca^2+^ sensors with greater dynamic ranges increases the possibility of exploring Ca^2+^ dynamics at the subcellular level and, notably, the interconnection between Ca^2+^ releases from different compartments. As such, the relation between cytoplasmic and nuclear Ca^2+^ signals, which remains unclear at the cellular level *in planta*, can now be addressed with increased resolution. Thus, we took advantage of the separate fluorescence emission windows of G-GECO (500–550 nm) and R-GECO (600–643 nm) to simultaneously assess Ca^2+^ dynamics in those contiguous cellular compartments. By targeting each of them to either the cytoplasm or the nucleoplasm, we developed the new dual sensors, NRCG-GECO1, NGCR-GECO1, and NRCG-GECO1.2 that allowed the simultaneous monitoring of nuclear and cytoplasmic Ca^2+^ dynamics in *M. truncatula* and *A. thaliana* root cells in response to biotic and abiotic stimuli. Despite the different association rates (K_on_) of R-GECO and G-GECO (Zhao et al., 2011), these values are in the order of 10^9^-10^15^ M^−1^·s^−1^, which means that binding of either sensor to Ca^2+^ occurs faster than image capture. Therefore, any differences observed in the response times between the nucleus and the cytosol are intrinsically biological.

In legume symbioses, nitrogen-fixing rhizobial NF and mycorrhizal factors induce Ca^2+^ oscillations in perinuclear and nuclear compartments. Although the importance of cytoplasmic Ca^2+^-mediated signal transduction is unknown, nuclear-localized Ca^2+^ is essential to activate a nuclear-localized Ca^2+^ and calmodulin-dependent kinase (CCaMK), that assures downstream transcriptional regulation of endosymbiotic programs (Zipfel and Oldroyd, 2017). Due to the lack of resolution, nuclear Ca^2+^ oscillations appeared synchronized between the nucleoplasm and the adjacent cytoplasmic region (Capoen et al., 2011). Furthermore, the ion channels and pump required to generate the symbiotic factor-induced Ca^2+^ oscillations were reported to localize to both the INM and ONM, although preferential localization of DMI1 to the INM was demonstrated (Capoen et al., 2011; Charpentier et al., 2016). Thus, the nuclear Ca^2+^ oscillations could start simultaneously or specifically from either nuclear membrane (Capoen et al., 2011). Using the NRCG-GECO1 and NGCR-GECO1 sensors, we demonstrated that Ca^2+^ release initiates inside the nucleoplasm in the majority of *M. truncatula* root hair cells elicited by NFs. This result unravels that the ion channels are activated first at the INM. This further suggests that the secondary messenger activating CNGC15 or DMI1 must either diffuse via the nucleopores or be produced inside the nucleus. Several nucleoporin mutants have been shown to be impaired in the generation of the nuclear Ca^2+^ oscillations in a temperature sensitive manner (Kanamori et al., 2006; Saito et al., 2007; Groth et al., 2010). However, their precise roles in this regulation remains unclear. Although the nucleoporins have been proposed to regulate the trafficking of the ion channels to the INM, our results suggest that they might be involved in the trafficking of the secondary messenger or proteins required for the activation of nuclear calcium oscillations.

Our studies further revealed the advantage of the dual GECO sensor to monitor subcellular Ca^2+^ dynamics in adjacent cytoplasm and nucleoplasm compartments of *A. thaliana* cells. By using a stably transformed dual sensor line we compared the dynamics of Ca^2+^ simultaneously in the cytoplasm and nucleoplasm of epidermal cells of the root elongation zone in response to diverse stimuli. We demonstrated that different stimuli (CO8, NaCl, ATP and cold shock) can generate, within the same cell type, diverse Ca^2+^ responses in the cytoplasmic and nucleoplasmic compartments. Notably, CO8 and ATP trigger cytoplasmic-associated oscillatory Ca^2+^ releases that do not diffuse to the nucleoplasm, suggesting that Ca^2+^ buffering proteins or Ca^2+^ uptake mechanisms are rapidly recruited or activated, respectively, to avoid Ca^2+^ diffusion into the nucleus. Interestingly, the ATP-induced Ca^2+^ signals in root cells of the elongation zone differ from a previous study showing dual cytoplasmic and nuclear Ca^2+^ releases (Krebs et al., 2012). This discrepancy might be due to the analysis of distinct root cells displaying specific ATP-induced Ca^2+^ responses, as the same stimulus can elicit different cell type-specific Ca^2+^ signatures (Kiegle et al., 2000). These findings further highlight the importance of monitoring Ca^2+^ dynamics in the same cell type to aptly compare stimuli-induced Ca^2+^ signatures and correlate the Ca^2+^ signature to a biological response. In contrast to CO8 and ATP, cold- and NaCl-induced transient Ca^2+^ signals first in the cytoplasm and then in the nucleoplasm. However, the NaCl- and cold-induced Ca^2+^ signatures in both compartments differ in amplitude and timing of activation, suggesting the involvement of different channels and/or pumps. Notably, the NaCl-induced nucleoplasmic Ca^2+^ signal was observed earlier (within 2 s) than the cold-induced Ca^2+^ signal (within 25 s), following the cytoplasmic Ca^2+^ release. Moreover, The NaCl-induced nucleoplasmic Ca^2+^ signal had a higher amplitude than the cytoplasmic Ca^2+^ signal, which contrasts with the cold-induced Ca^2+^ signal, smaller in amplitude. These results support the induction of nuclear Ca^2+^ signals independently of the cytoplasmic Ca^2+^ release, as implied by a recent study regarding the NaCl-induced cytoplasmic/nuclear Ca^2+^ dynamics (Huang et al., 2017). Alternatively, the cytoplasmic Ca^2+^ release might be contributing to the generation of the nuclear Ca^2+^ signal either by diffusion or by triggering a Ca^2+^-induced Ca^2+^ release mechanism at the nuclear envelope.

In summary, the cytoplasmic and nucleoplasmic dual sensors developed in this work are powerful tools to analyze the subcellular Ca^2+^ dynamics between the cytoplasm and nucleus *in planta*. These sensors will unequivocally be of interest in exploring Ca^2+^-mediated signaling in response to diverse stimuli in different plant species.

## Materials and methods

### DNA constructs

The single and dual GECO Ca^2+^ sensor constructs (Figure S1) were assembled using Golden Gate cloning. All GECO1.0 sensors were cloned under the control of the double cauliflower mosaic virus *35S* promoter (*2xp35S*) and the 35S terminator in the Golden Gate compatible vector *pCambiaCR1* Δ*DsRed* (Fliegmann et al., 2016) kindly provided by C. Rosenberg (LIPM). PCR amplification of the individual sequence modules was done using Phusion Taq high fidelity DNA polymerase (New England Biolabs) and respective DNA templates and primer pairs are listed in Table S1. Amplified DNA fragments flanked by *Bsa*I and specific cohesive protruding ends were cloned into pBlueScript II (Agilent) and validated by sequencing before being used in Golden Gate assembly reactions (Engler and Marillonnet, 2014). All level 0 modules used in the dual NRCG-GECO1.2 sensor were synthesized by Life Technologies™ (ThermoFisher Scientific). All assembled Golden Gate binary vectors were verified by PCR, DNA sequencing and restriction digestion before transformation into *Agrobacterium*. The cameleon Ca^2+^ sensors *NUP-YC2.1* and *NLS-YC3.6* used here were previously generated in Sieberer et al. (2009) and Krebs et al. (2012).

### Plant material and bacterial strains

Four *M. truncatula* cv Jemalong A17 lines were used in this study: the wild type and the symbiotic mutants *nfp-2* (Arrighi et al., 2006) *dmi1*-*1* and *dmi3*-*1* (Catoira et al., 2000; Wais et al., 2000). *M. truncatula* seeds were scarified with sulfuric acid, then surface-sterilized prior to germination and grown on inverted soft Campbel agar plates (http://www.noble.org/medicagohandbook). Arabidopsis seeds were surface-sterilized in 1.5% bleach for 15 min, followed by five washes in sterile water, and then plated in Murashige and Skoog (MS) solid medium (1% sucrose). After 3–5 days at 4°C, plates were moved to a growth cabinet (23°C, 16-h photoperiod, and 300 μmol·m^−2^·s^−1^ light intensity) for germination and growth. The *Escherichia coli* DH5α strain was used for plasmid propagation, the *A. rhizogenes* Arqua1 strain (Quandt et al., 1993) strain for the generation *M. truncatula* composite plants and the *A. tumefaciens* GV3101 (Koncz and Schell, 1986) strain for stable transformation of *A. thaliana*.

### Generation and *in vitro* culture of *Agrobacterium*-transformed *M. truncatula* and *A. thaliana*

*A. rhizogenes*-mediated transformation hairy root transformation of *M. truncatula* was performed as described in Boisson-Dernier et al. (2001) with minor modifications. Germinated seedlings were sectioned ~3 mm from the root tip and inoculated with a 3–4 μL drop of *A. rhizogenes* bacterial suspension adjusted to an OD_600nm_ of 1 in water. Inoculated seedlings were placed on Fahraeus/agar plates supplemented with 0.5 mM ammonium nitrate and kanamycin (25 mg.L^−1^). Plates were partially sealed with parafilm to allow crucial gas exchanges and placed in a 20°C growth chamber (16-h photoperiod and a light intensity of 70 μE·m^−2^·s^−1^) for 1 week before transferring to 25°C. About 2 weeks-post-inoculation, kanamycin-resistant composite plants were transferred to a beaker containing sterile water supplemented with 200 mg·L^−1^ augmentin (amoxicillin:clavulanic acid [5:1], GlaxoSmithKline), and incubated for at least 30 min, in order to reduce the growth of *A. rhizogenes*. Plants were rinsed twice before being transferred to square plates containing a nitrogen-free modified Fahraeus medium with 0.5% [w/v] phytagel (Sigma-Aldrich) and supplemented with 50 nM 2-amino-ethoxyvinyl-Gly (AVG), adapted for *in vivo* microscopic observations as described in Fournier et al. (2008). Transgenic roots were covered with a sterile, gas-permeable and transparent plastic film (Lumox film, Starsted) that allows the use of water-immersion objectives during imaging. Square plates with composite plants were slightly tilted to encourage the growth of the roots along the plastic film. The lower part of the square plate was wrapped in black plastic to protect the roots from light. *M. truncatula* ROCs expressing the NR-GECO1 sensor were obtained from transgenic composite plants as described by Boisson-Dernier et al. (2001). ROCs were cultured on M medium (Boisson-Dernier et al., 2001) and propagated vertically until used for microscopy observations.

*A. thaliana* stable transgenic plants were generated using the floral dip method (Clough and Bent, 1998) and selected in 50 μg·mL^−1^ hygromycin B.

### Selection and symbiotic treatments of *M. truncatula* transformed roots

Kanamycin-resistant *A. rhizogenes*-transformed composite plants or ROCs transformed with cameleon or GECO1 constructs were screened for expression of the respective fluorescent sensors using fluorescence stereomicroscopes (Leica MZFLIII and Zeiss axiozoom V16) and a Zeiss Axiophot epifluorescent microscope. Selection of transgenic roots was done using filters adapted to detect yellow/green YFP/GFP (excitation: 470–510 nm; emission: 525–575 nm) or red mApple (excitation: 577–587 nm, emission: 620–680 nm) fluorescent proteins from the different cameleon or GECO1 sensors. Only composite plants with fluorescent transgenic roots were transferred to plates containing nitrogen-free modified 0.5% [w/v] phytagel Fahraeus medium for imaging 3–5 days later. Transgenic roots lying on the medium and covered by a gas-permeable film were treated with 1 to 2 mL of a freshly diluted aqueous solution of purified *S. meliloti* NFs (10^−9^ M) or CO4 (10^−5^ M or 10^−7^ M) that were added to plates between the plastic film and the medium with the help of a micropipette. In the case of ROCs, imaging was performed with excised root sections that were mounted between a slide and a cover slip. Confocal imaging was performed before treatments to assess the background fluorescence levels, and then initiated about 5 min after treatment for a total period of up to 1 h.

### Confocal imaging of calcium responses in transgenic *M. truncatula* and arabidopsis roots

The comparison between the nuclear NR-GECO1, the NUP-YC2.1 and the NLS-YC3.6 Yellow Cameleon sensors was performed using a Leica TCS SP2 AOBS confocal laser-scanning microscope equipped with a long-distance 40x water-immersion objective (HCX Apo L 0.80). The 458 nm argon laser was used to excite the CFP of the cameleon sensors (pinhole diameter at 4 Airy units). Fluorescence emission was collected in the 470–500 nm range for CFP and 530–570 nm range for YFP. Images were acquired at 5 s intervals from 5 to 60 min either before or after treatment with a 512 × 512 pixels resolution. The 561 nm diode was used to excite the mApple red fluorescence protein of the NR-GECO1 sensor (pinhole diameter at 3 Airy units) and fluorescence was visualized in the specific 580 to 643 nm emission window. The laser intensity used for the cameleon probes (80% power setting, Sieberer et al., 2009) was reduced to 20% for monitoring Ca^2+^ responses with the NR-GECO1 sensor in order to avoid photo bleaching of the red fluorescence during long acquisitions. Confocal imaging of transgenic roots expressing the dual NRCG-GECO1 and NGCR-GECO1 sensors was done using a beam-splitter Leica TCS SP8 AOBS confocal laser-scanning microscope equipped with a 25x water-immersion objective. The 488 nm argon laser was used to excite the G-GECO1 and the 561 nm diode to excite the R-GECO1. Fluorescent images were recorded simultaneously for NRCG-GECO1 and NGCR-GECO1, using emission windows of 500–550 nm and 600–643 nm for G-GECO1 and R-GECO1 signals, respectively. Fluorescence emission ranges were specifically set for each fluorescent protein in order to avoid overlap between the G-GECO1 and R-GECO1 signals, as detailed in Figure S6. Images were acquired in 200 to 500 ms intervals with a scanning resolution of 512 × 128 pixel. Images were acquired using the Leica confocal software.

The subcellular localization of the GECO1.2 Ca^2+^ reporters was assessed in 6-day-old roots by CLSM using a Zeiss LSM 780 equipped with a 25x/0.8 water objective. G-GECO1.2 was excited at 488 nm and the emitted light was captured between 463 and 500 nm. R-GECO1.2 was excited at 561 nm and emitted fluorescence captured in the 571–640 nm range. For Ca^2+^ imaging, plants were analyzed 5–6 days after germination and carefully mounted in a perfusing (cold treatment) or non-perfusing chamber. Perfusion of cold media was performed using a RC-21BR chamber (Warner Instruments). For ATP, CO8, and NaCl treatments, the elicitors were applied at a 10x concentration to an open chamber made with cover glass and sealed with vacuum grease. After mounting in liquid MS (1% sucrose), samples were incubated at room temperature for at least 15 min before imaging. Ca^2+^ imaging was performed using a Nikon ECLIPSE FN1. The fluorophores were excited at a wavelength of 470 ± 24 nm. Emitted fluorescence was separated by an image splitter and passed through an emission filter of 520 ± 40 nm for G-GECO1.2, and 632 ± 60 nm for R-GECO1.2 (Optosplit, Cairn Research, UK). Images were collected every 2 or 3 s.

### Image analysis

Processing, final cropping, and mounting of the Medicago images were performed using Fiji. The fluorescence, merged images and Supplementary time-lapse movies were obtained after applying a median filter of 2 pixel radius to fluorescence image series. Intensity data was calculated from selected ROI, delimited in individual root hairs within the nuclear region (for nuclear-localized sensors) or tailored in the perinuclear, middle or root hair tip regions (for cytoplasmic sensors). To quantify relative Ca^2+^ spiking amplitudes, the average fluorescence for each ROI was calculated and used to determine the SNR of individual spikes collected from independent roots and biological experiments. The SNR of a given spike measures the difference between the fluorescence baseline and the maximum fluorescence peak value. The SNR values of individual spikes were obtained by calculating the ΔF/F (F_max_-F_min_/F_min_) or ΔR/R (R_max_-R_min_/R_min_), in which F_max_ and R_max_ correspond to the maximum fluorescence value or ratio change of a spike and F_min_ and R_min_ correspond to the data point before the onset of a spike.

For image processing of the Arabidopsis data the following steps were conducted using ImageJ 1.48v: background subtraction, registration using MultiStackReg v1.45 (http://bradbusse.net/sciencedownloads.html), and application of a lookup table. Image data were obtained from processed images using Time Series Analyzer V3_2 (https://imagej.nih.gov/ij/plugins/time-series.html). Normalized datasets (ΔF/F) were calculated as (F–F_0_)/F_0_, where F_0_ represent the average of at least 2 min of baseline values (before treatment).

### Statistical analyses

Statistical analyses were performed using R (http://r-project.org). Data were evaluated for normality with the Shapiro-Wilk test and homogeneity of variance with Levenne or Bartlett tests. Parametric (ANOVA) or non-parametric (Kruskal-Wallis) statistical tests were used, respectively, for normally-distributed data and non normally-distributed data. Signal periodicity values of calcium sensors were first transformed into normality using Box-Cox (λ = 0.0606), before statistical analysis using one-way ANOVA followed by Tukey honest difference (HSD) tests. Statistical analysis of signal-to-baseline ratio was done using the non-parametrical Kruskal-Wallis test. For the dual GECO sensors, statistical analyses of the start of a calcium spike was done using a chi-square test (*p* < 0.05) to (i) compare the frequency of observations in each category to an expected frequency of 1/3 per category if the distribution was random and (ii) to compare the number of observations per category for each sensor grouped in a contingency table.

## Author contributions

MyC and FdC-N: conceived the project; AK, NL, MyC, and FdC-N: designed the experiments; AK: performed the *M. truncatula* and NL: the *A. thaliana* experiments; MiC: contributed to the NLS-R-GECO1 in ROCs experiments; AK and NL: prepared the figures; AK, NL, MyC, and FdC-N: wrote the manuscript; MyC and FdC-N: are co-responsible for the work.

### Conflict of interest statement

The authors declare that the research was conducted in the absence of any commercial or financial relationships that could be construed as a potential conflict of interest.
