# Supplementary material for: Dual Color Sensors for Simultaneous Analysis of Calcium Signal Dynamics in the Nuclear and Cytoplasmic Compartments of Plant Cells
Source: Front Plant Sci. 2018 Feb 27;9:245. doi: 10.3389/fpls.2018.00245 (PMC5835324; doi:10.3389/fpls.2018.00245)
Supplement: Supplementary file 7 [file DataSheet1.PDF]

Supplementary Material

Dual colour sensors for simultaneous analysis of calcium signal dynamics in the nuclear and cytoplasmic compartments of plant cells

Audrey Kelner†, Nuno Leitão†, Mireille Chabaud, Myriam Charpentier* and Fernanda de Carvalho-Niebel*

† Equal contributions

*** Correspondence:**

Myriam Charpentier (myriam.charpentier@jic.ac.uk)

Fernanda de Carvalho-Niebel (fernanda.de-carvalho-niebel@inra.fr)

1 Supplementary Tables

Name	Sequence (5'-3')
**AB-**2x35Sp-GGfw	GGTCTCT**AAAT**AAGCTTGCATGCCTGCAGGTC
**AB-**2x35Sp-GGrev	GGTCTCA**TTTG**TGTTTTTTTCGGTACCCCGGG
**BD**-NLS-R-GECO_GGfw	GGTCTCT**CAAA**ATGGACCCAAAAAAGAAGCG
**BD**-NLS-R-GECO_GGrev	GGTCTCC**CGTA**CTACTTCGCTGTCATCATTTGTAC
**BD**-G-GECO1_GGfw	GGTCTCC**CAAA**ATGGTCGACTCATCACGTCGT
**BD**-G-GECO1_GGrev	GGTCTCA**CGTA**CTACTTCGCTGTCATCATTTG
**BX**-NLS-RG-T35S-GGfw	GGTCTCT**CAAA**ATGGACCCAAAAAAGAAGCG
**BX**-NLS-RG-T35S-GGrev	GGTCTCC**CTGG**AATTAGAAATTTTATTGATAGAAG
**BZ**-NLS-GGfw	GGTCTCT**CAAA**ATGGACCCAAAAAAGAAGCG
**BZ**-NLS-GGrev	GGTCTCC**ATCA**CCTTGCGTTTCTTCTTAGGATC
**ZX**-GGm-T35S-GGfw	GGTCTCG**TGAT**GGTCGACTCATCACGTCGTAAG
**ZX**-GGm-T35S-GGrev	GGTCTCC**CTGG**AATTAGAAATTTTATTGATAGAAG
**XC-**2x35Sp-GGfw	GGTCTCT**CCAG**AAGCTTGCATGCCTGCAGGTC
**XC-**2x35Sp-GGrev	GGTCTCA**CACC**TGTTTTTTTCGGTACCCCGGG
**CY**-G-GECO1_GGfw	GGTCTCA**GGTG**GAATGGTCGACTCATCACGTCGT
**CY**-G-GECO1_GGrev	GGTCTCG**GGAC**CGCATCACCGTCCCCAGCTCCT
**YD**-G-GECO1_GGfw	GGTCTCG**GTCC**CTGGGGCAGAACCCCACAGAAG
**YD**-G-GECO1_GGrev	GGTCTCA**CGTA**CTACTTCGCTGTCATCATTTG
**CW-**NES-GGfw	GGTCTCT**GGTG**GAATGCTGCAGAACGAGCTTG
**CW**-NES-GGrev	GGTCTCA**ATGG**TGGCGGCCGCACTCGAGTCGA
**WD**-R-GECO-GGfw	GGTCTCT**CCAT**GGTCGACTCATCACGTCGTAA
**WD**-R-GECO-GGrev	GGTCTCG**CGTA**CTACTTCGCTGTCATCATTTG

TABLE S1 | Golden-Gate sequence blocks and primers used in this study.

List of primers used to PCR amplify the different Golden Gate sequence blocks with the Phusion Taq high fidelity DNA polymerase. Nucleotides in bold correspond to the fusion sites and *Bsa*I sites are underlined.

2 Supplementary Figures

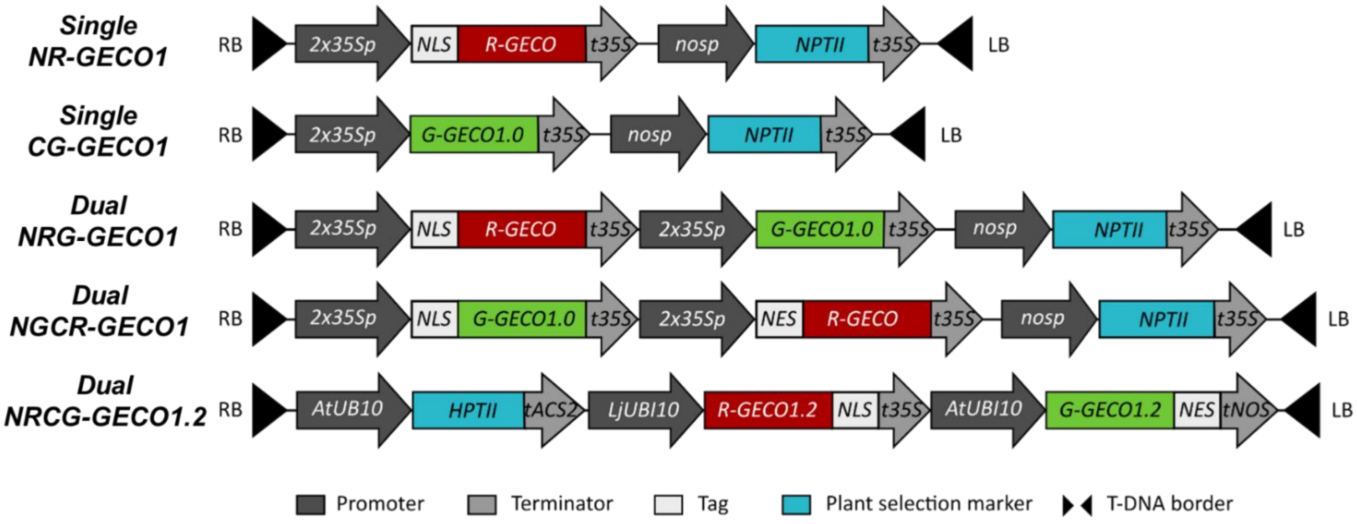

FIGURE S1 | Golden-Gate cloning strategy used to generate single and dual GECO sensors expressed in *M. truncatula*.

In NR-GECO1, G-GECO1, NRCG-GECO1 and NGCR-GECO1 vectors, the GECO1 coding sequences are expressed under the control of the *CAMV 35S* promoter (*2x35Sp)* and the *35S* terminator (*t35S*). GECO1 modules have a nuclear localization signal (NLS) or a nuclear exclusion signal (NES) in the 5' end, as indicated. In NRCG-GECO1.2, the R-GECO1.2 expression cassette is flanked by the *L. japonicus* UBIQUITIN10 (LjUBI10) promoter and t35S and features an NLS at the 3' end. The G-GECO1.2 coding sequence is flanked by the Arabidopsis UBIQUITIN10 (AtUBI10) promoter and the *NOPALISE SYNTHASE (NOS)* terminator (tNOS) and features a nuclear exclusion signal (NES) at the 3' end. These vectors have different plant selection genes, *NPTII* and *HPTII*, conferring resistance to kanamycin and hygromycin B, respectively. Expression of *NPTII* is controlled by the *NOS* promoter and t35S, and expression of *HPTII* is driven by the AtUBI10 promoter and the terminator for 1-*AMINOCYCLOPROPANE-1-CARBOXYLATE SYNTHASE 2* (*ACS2*). Regulatory and coding elements are indicated by the colour code.

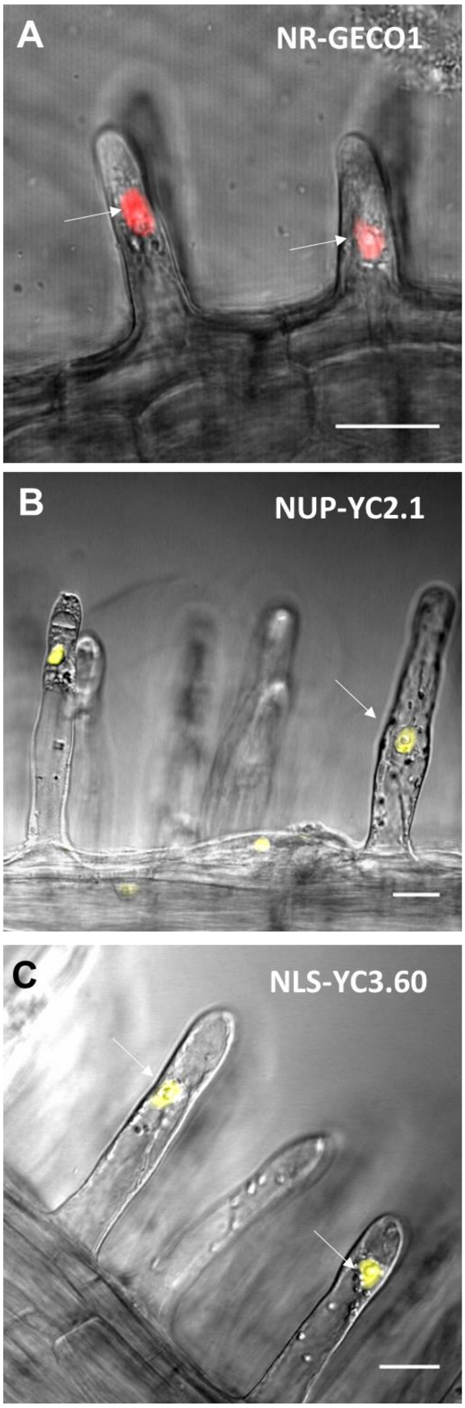

FIGURE S2 | NR-GECO1, NUP-YC2.1 and NLS-YC3.6 Ca^{+2} sensors are targeted to the nuclear compartment in transgenic *M. truncatula* root hair cells.

(A-C) Merged fluorescent and bright field images of NR-GECO1 **(A)**, NUP-YC2.1 **(B),** and NLS-YC3.6 **(C)** in transgenic *M. truncatula* root hair cells (white arrows). Scale bars represent 20 µm.

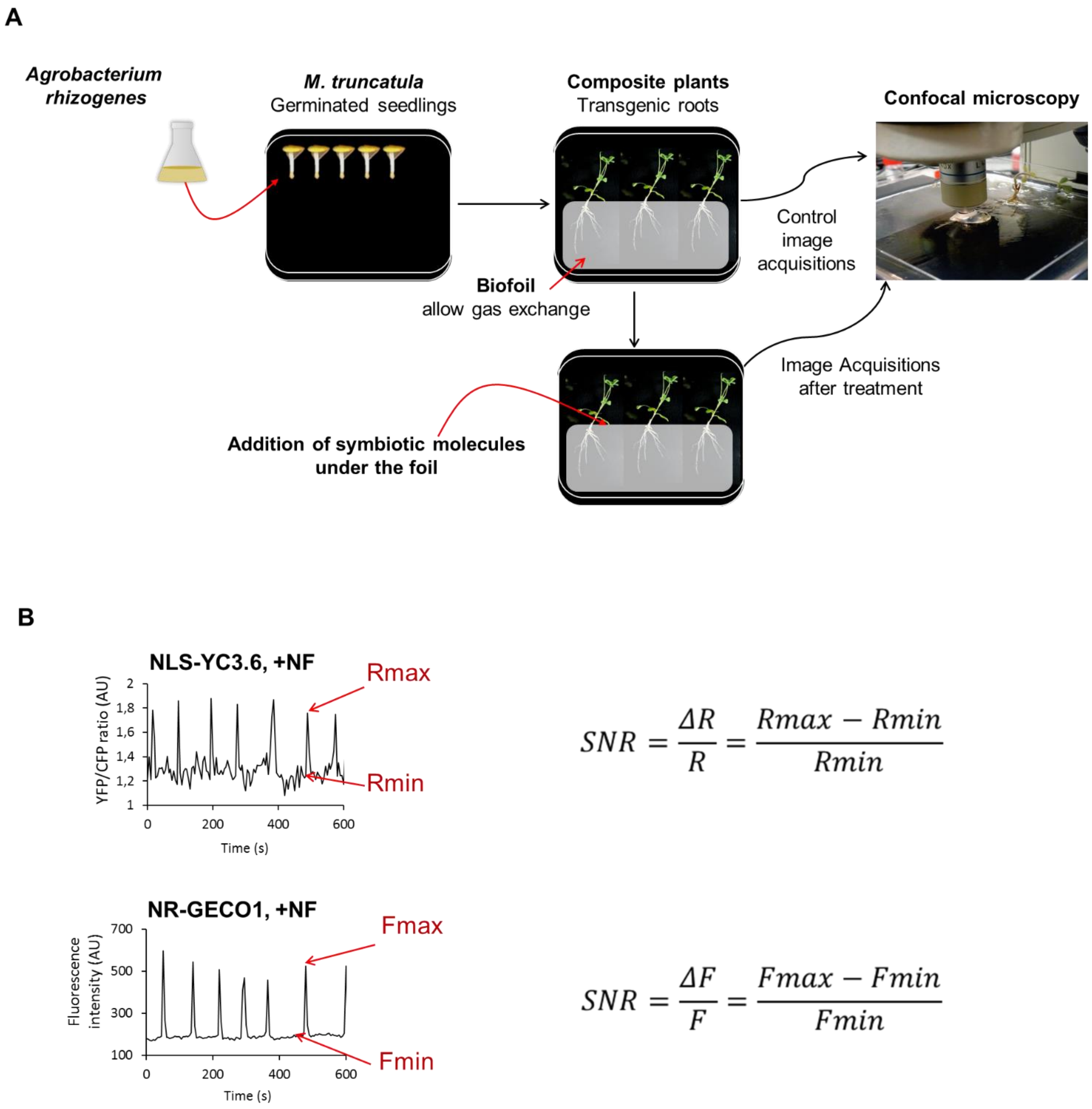

FIGURE S3 | Methodology used to measure symbiotic calcium responses in *M. truncatula* expressing different calcium sensors.

(A) Description of the different stages of growth and treatments of *M. truncatula* composite plants before confocal imaging. **(B)** Schematic representation the fluorescence intensity or ratio values used to calculate respective SNRs.

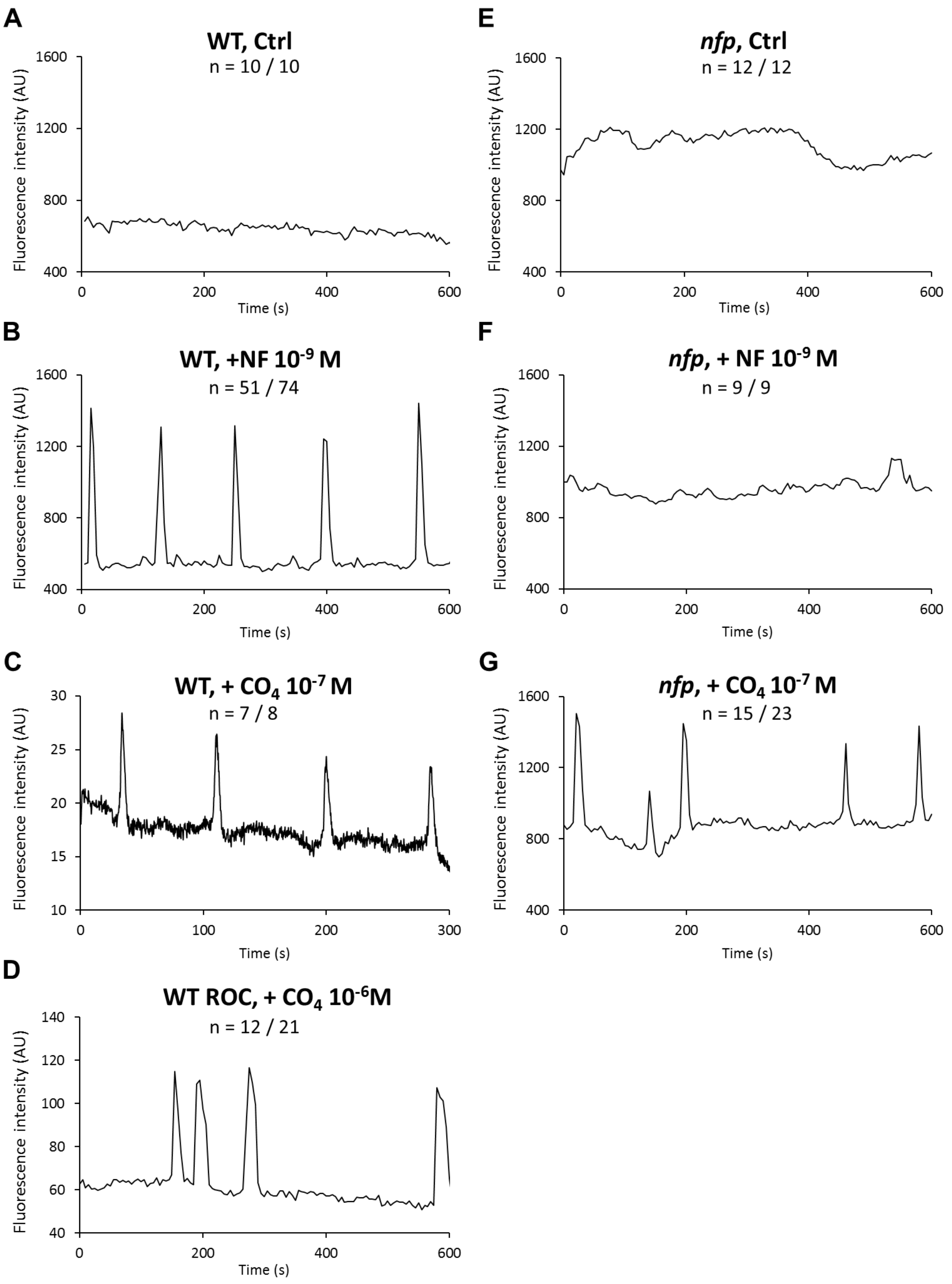

FIGURE S4 | Differential Ca^{2+} responses in the *nfp* symbiotic mutants using NR-GECO1.

Representative Ca^{2+} spiking traces of WT **(A-C)** and *nfp* **(E-G)** mutant root hairs expressing NR-GECO1 in the absence of treatment (Ctrl) **(A, E)**, in response to 10^{-9} M Nod Factors (+NF) **(B, F)** and in response to 10^{-7} M CO4 (+CO4) **(C, G)**. **(G)** Representative trace of a wild-type root organ culture (ROC) epidermal cell transformed with NR-GECO1 responding to 10^{-7} M CO4. n represents the number of root hair cells responding/root hair cells analysed. Data were obtained from 1 **(C-G)** or 2 biological experiments **(A-B)**.

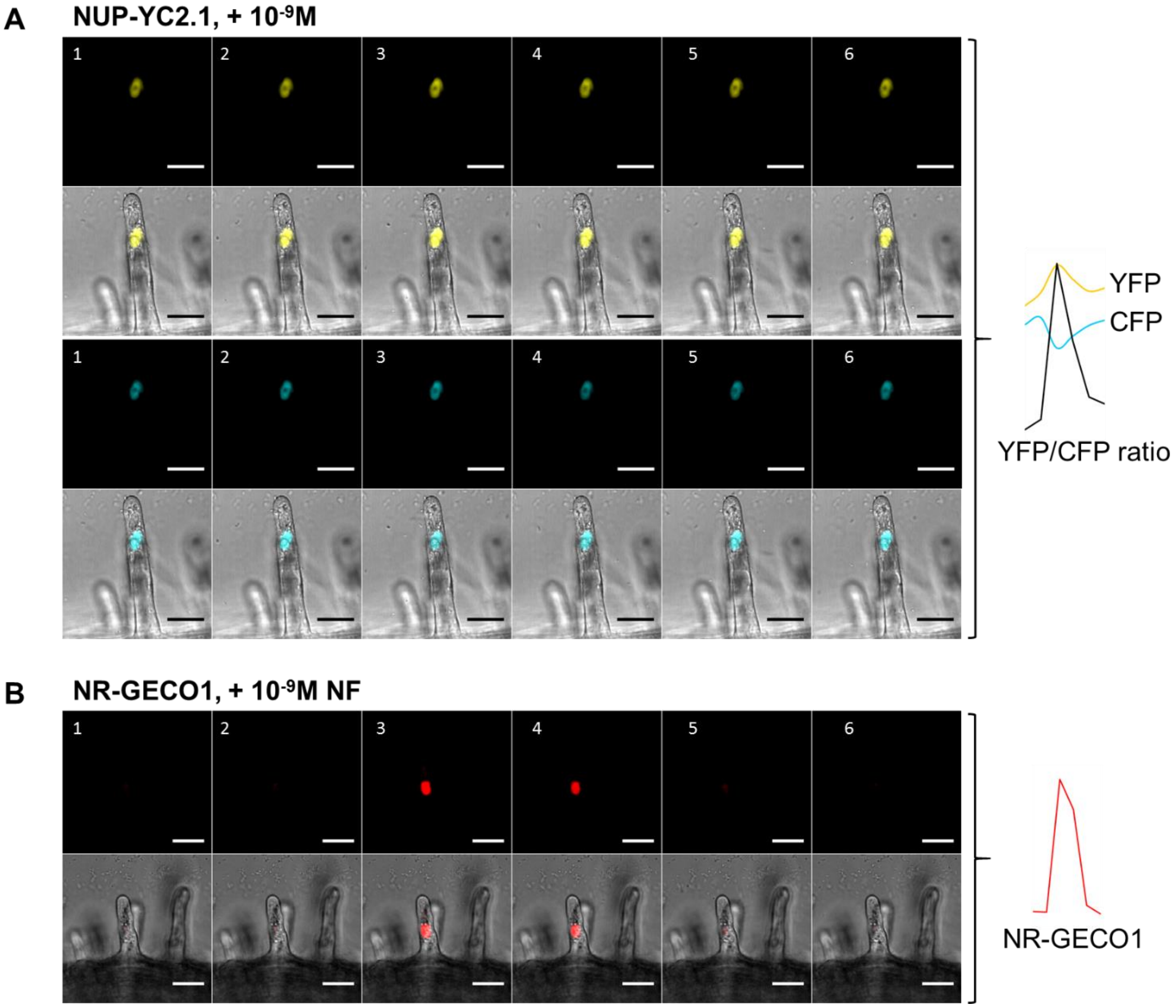

FIGURE S5 | Direct monitoring of a Ca^{2+} spike using the high signal-to-noise ratio NR-GECO1 sensor.

Fluorescence and merged fluorescence/bright field confocal images taken every 5s of single spikes with the NUP-YC2.1 cameleon probe (**A**) and NR-GECO1 (**B**). The corresponding fluorescence intensity (for NR-GECO1 and NUP-YC2.1) and YFP to CFP ratio (for NUP-YC2.1) spiking profiles during a 30 s interval are graphically represented on the right side. Scale bars represent 20 µm.

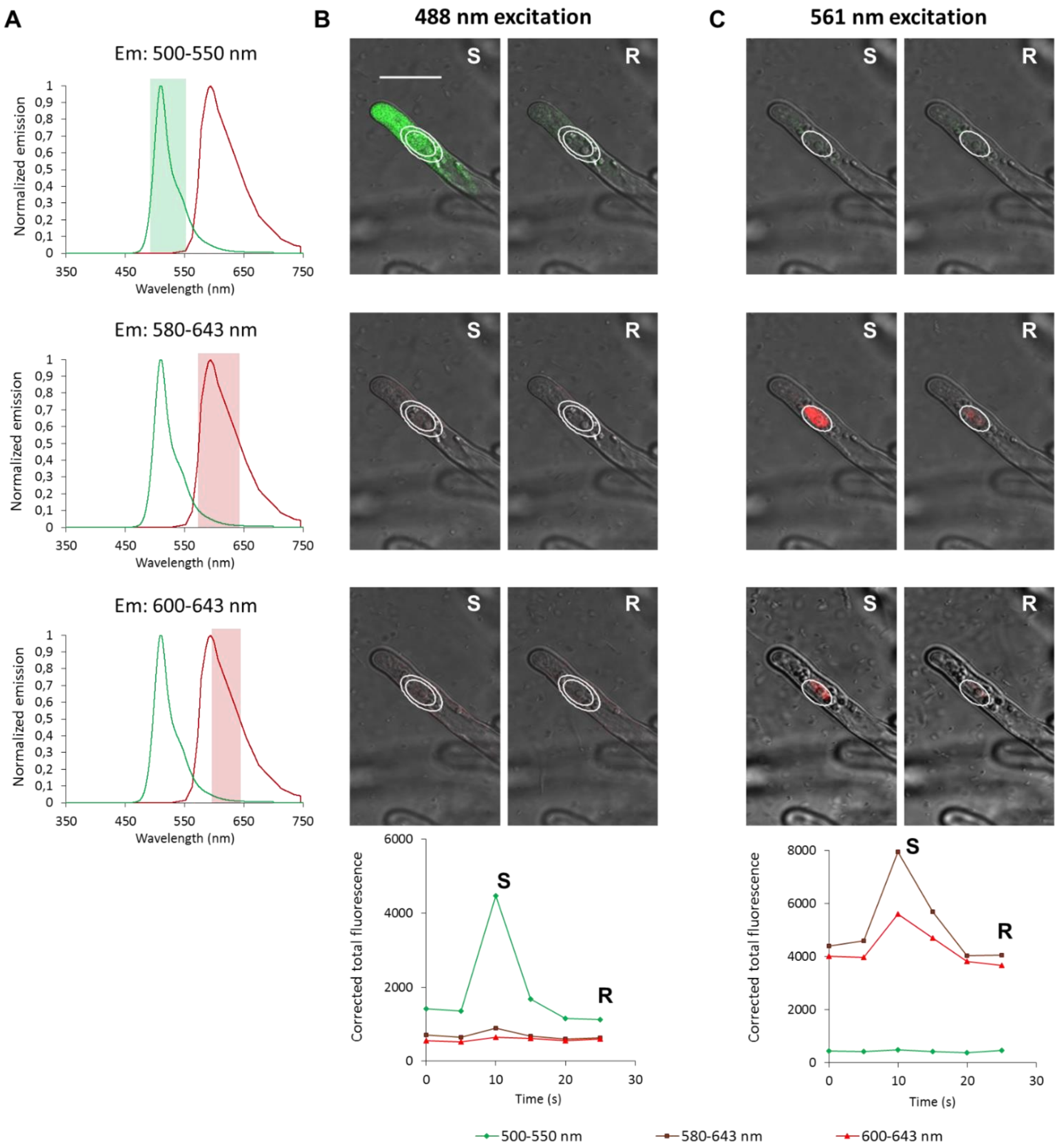

FIGURE S6 | Fluorescence acquisition parameters for co-imaging nuclear NR-GECO1 and cytoplasmic CG-GECO1 in *M. truncatula* root hair cells expressing the dual sensor.

Crossed excitation and emission of the two sensors were used here to examine possible overlaps between the emission fluorescence of the two sensors. Composite bright-field/fluorescent images of a root hair treated with 10^{-9} M NF after excitation at 488 nm **(B)** or 561 nm **(C)**. The images show the relative fluorescence obtained in different emission (Em) windows illustrated in **(A)**, with the emission spectra of the different fluorescent proteins, during a spike (S, left images) or in a resting situation (R, right images). Corrected total fluorescence was calculated using the formula CTF = Integrated density – (ROI Area x Mean fluorescence of background) using Fiji. Scale bars represent 20 µm.

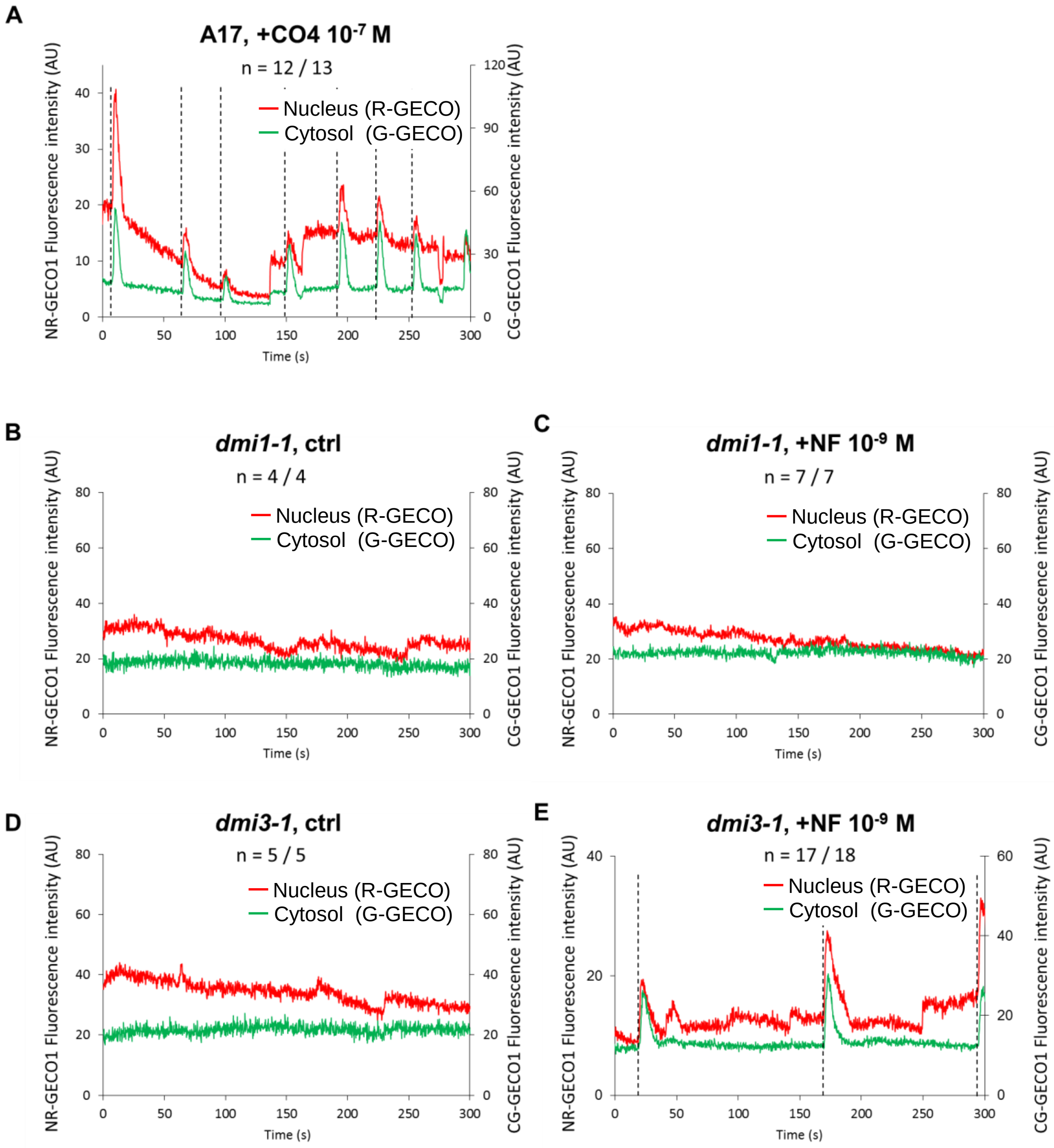

FIGURE S7 | Simultaneous detection of nuclear and cytoplasmic Ca^{2+} responses in symbiotic mutants of *M. truncatula* using the double NRCG-GECO1 sensor.

Representative Ca^{2+} traces of NR-GECO1 (red) and CG-GECO1 (green) at 0.25 s intervals in WT **(A)**, *dmi1-1* **(B-C)** and *dmi3-1* **(D-E)** root hairs in control (ctrl) **(B, D)**, and treated with 10^{-9} M Nod Factor (+NF) **(C, E)** or 10^{-7} M CO4 **(A)**. n corresponds to the number of root hairs responding positively/total number of analysed root hairs. Data were collected from 1 biological experiment **(A-E)**.

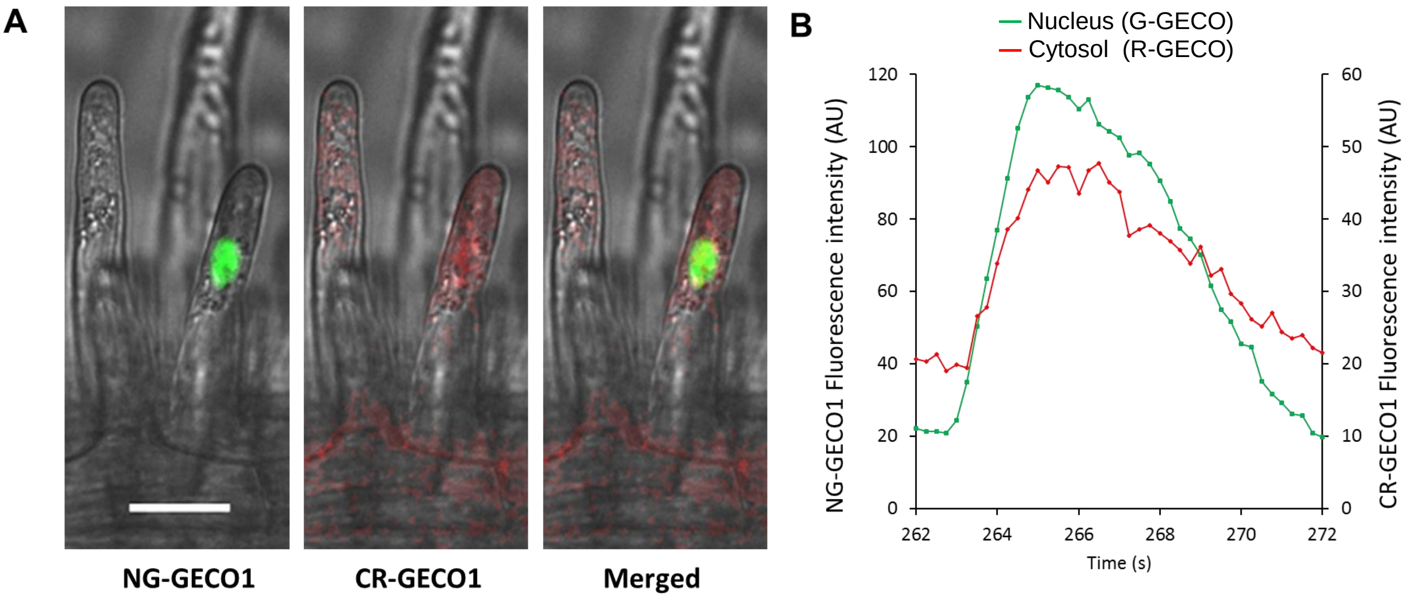

FIGURE S8 | The dual NGCR-GECO1 calcium sensor expresses G-GECO1 and R-GECO1, respectively, in the nuclear and cytoplasmic subcellular compartments.

(**A**) Confocal image of NGCR-GECO1 sensor during a Ca^{2+} spike in a *M. truncatula* root hair (green, red, and merged images with respective bright fields). Scale bar represents 20 µm. **(B)** Representative trace during a Ca^{2+} spike of a root hair cell (0.25 s intervals) expressing the dual NGCR-GECO1 sensor and treated with 10^{-9} M Nod Factors (+NF).

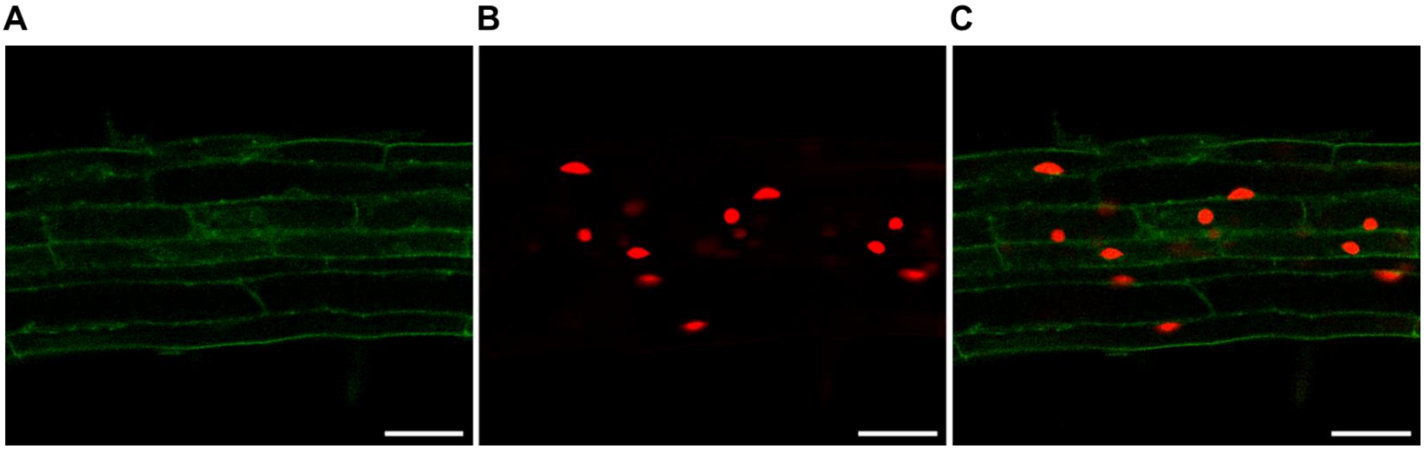

FIGURE S9 | Subcellular localization of the NRCG-GECO1.2 Ca^{2+} sensor in Arabidopsis roots.

Analysis of the elongation zone of Arabidopsis roots (6 days after germination) expressing the nuclear-excluded G-GECO1.2 and the nuclear-localized R-GECO1.2. Green channel **(A)**, red channel **(B)**, merged **(C)**. Scale bars represent 50 µm.

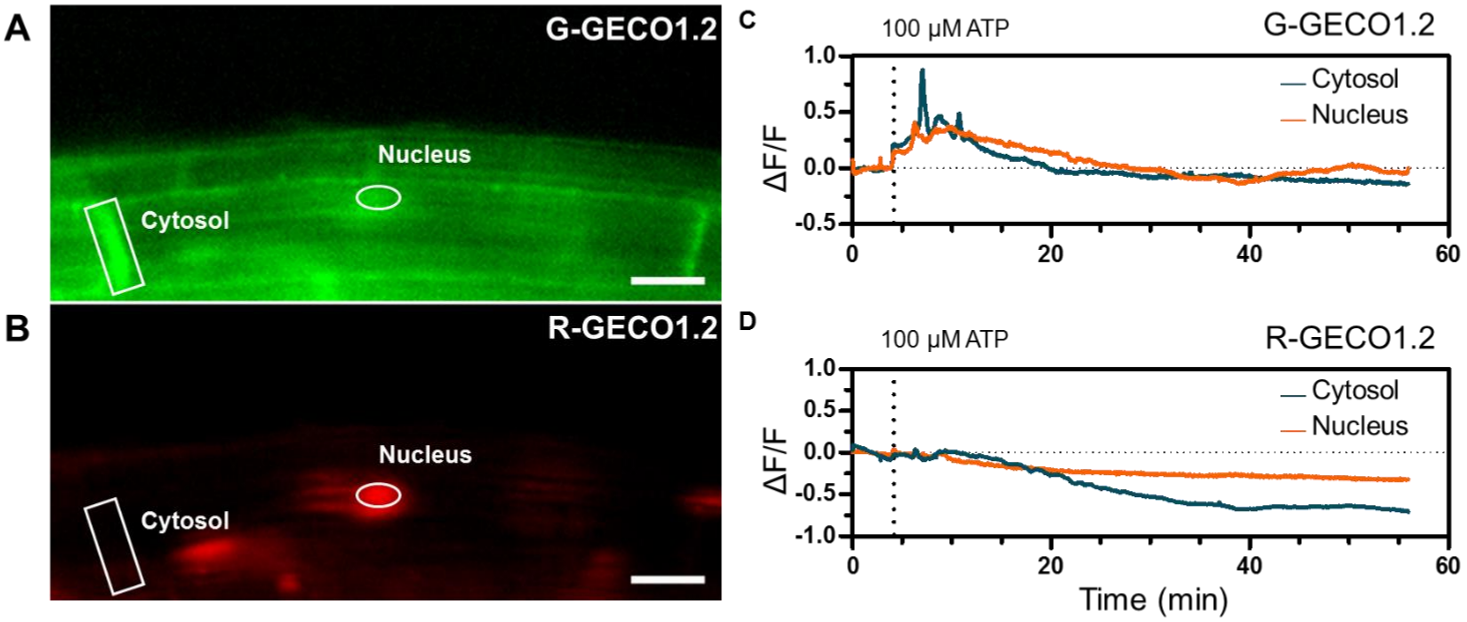

FIGURE S10 | The dual Ca^{2+} sensor reports Ca^{2+} dynamics to ATP application.

Ca^{2+} signals induced by 100 µM ATP in a 5-day-old root (elongation zone) expressing the dual Ca^{2+} reporter NRCG-GECO1.2 (G-GECO1.2 channel **(A)**, R-GECO1.2 channel **(B)**). **(C-D)** Normalised fluorescence intensities in the G-GECO1.2 channel **(C)** and the R-GECO1.2 channel **(D)** in the cytoplasm (blue) and the nucleus (orange) (ROIs marked in **(A)** and **(B)**). Dashed vertical lines mark the moment of ATP application (n = 5 plants). Scale bars represent 50 µm.

3 Supplementary Movies

MOVIE S1 | Direct visualization of NF-elicited nuclear Ca^{2+} spiking in *M. truncatula* root hairs and epidermal cells expressing the single NR-GECO1 sensor.

Time-lapse confocal analysis of NR-GECO1 fluorescent changes during sequential Ca^{2+} spikes in *M. truncatula* roots responding to 10^{-9} M NFs. Scale bar represents 20 μm. Time format is min:sec.

MOVIE S2 | Direct visualization of CO4-elicited Ca^{2+} spiking in epidermal cells of *M. truncatula* root organ cultures expressing the NR-GECO1 sensor.

Time-lapse confocal analysis of NR-GECO1 fluorescent changes during sequential Ca^{2+} spikes in ROCs responding to 10^{-6} M CO4. The movie corresponds to the measurement shown in **Supplemental Figure S4D**. White arrows indicate nuclei undergoing Ca^{2+} spiking. Scale bar represents 20 μm. Time format is min:sec.

MOVIE S3 | Simultaneous visualization of nuclear and cytoplasmic Ca^{2+} spiking in a *M. truncatula* root hair expressing the dual NRCG-GECO1 sensor in response to Nod Factor.

Time-lapse confocal analysis of NRCG-GECO1 fluorescent changes during sequential Ca^{2+} spikes in a *M. truncatula* root hair responding to 10^{-9} M NFs. Ca^{2+} spiking is visualized simultaneously in nuclear (in red) and cytoplasmic (green) compartments. Ca^{2+} spiking in the nuclear region is also seen in yellow due to the merged nuclear (in red) and peri-nuclear (in green) fluorescent signals. Movie corresponds to the measurement shown in **Figure 3B**. Scale bar represents 20 μm. Time format is min:sec:msec.

MOVIE S4 | Simultaneous visualization of nuclear and cytoplasmic Ca^{2+} spiking in a *M. truncatula* root hair expressing the dual NGCR-GECO1 sensor in response to Nod Factor.

Time-lapse confocal analysis of NGCR-GECO1 fluorescent changes during sequential Ca^{2+} spikes in a *M. truncatula* root hair responding to 10^{-9} M NFs. Ca^{2+} spiking is visualized simultaneously in nuclear (in green) and cytoplasmic (red) compartments. Ca^{2+} spiking in the nuclear region is also seen in yellow due to the merged nuclear (in green) and peri-nuclear (in red) fluorescent signals. Scale bar represents 20 μm. Time format is min:sec:msec.

MOVIE S5 | The nuclear and cytosolic Ca^{2+} signals in response to salt stress.

Ca^{2+} signals induced by treatment with 100 mM NaCl in a 6-day-old Arabidopsis root (elongation zone) expressing the dual Ca^{2+} reporter NRCG-GECO1.2. Time format is min:sec.

MOVIE S6 | The nuclear and cytosolic Ca^{2+} signals in response to cold.

Ca^{2+} signals induced by perfusion of cold medium in a 6-day-old Arabidopsis root (elongation zone) expressing the dual Ca^{2+} reporter NRCG-GECO1.2. Time format is min:sec.
